# Host–strain compatibility shapes host transcriptional responses in macrophage in vitro infection models of Mycobacterium tuberculosis

**DOI:** 10.1099/mgen.0.001826

**Published:** 2026-09-23

**Authors:** Paula Ruiz-Rodriguez, Marta Caballer-Gual, Guillem Santamaria, Hellen Hiza, Mireia Coscollá

**Affiliations:** Institute for Integrative Systems Biology (ISysBio), University of Valencia-CSIC, FISABIO Joint Research Unit Infection and Public Health, Valencia1, Spain; 2 Luxembourg Centre for Systems Biomedicine (LCSB), University of Luxembourg, Esch-Belval Esch-sur-Alzette, Luxembourg

**Keywords:** host–pathogen interactions, *Mycobacterium tuberculosis*, phospholipase, RNA-seq, transcriptomics

## Abstract

Differences in host distribution are observed across *Mycobacterium tuberculosis* complex (MTBC) lineages, but investigating the molecular basis of host compatibility *in vivo* is challenging. Here, we establish an epidemiology-guided *in vitro* framework to operationalize ‘host compatibility’ as a theoretical parameter that can be approximated experimentally using macrophage infection readouts. We infected two widely used but biologically distinct models, human THP-1-derived macrophages (THP-1m) and bovine macrophages (BoMac), with four MTBC human and animal strains. We quantified infected-cell frequency, intracellular bacterial burden dynamics, cell death and host transcriptome by RNA-seq. Across both models, infection phenotypes were concordant with the *a priori* epidemiology-informed reference pairings: the most compatible strain showed a higher late intracellular bacterial burden in its respective host, and the corresponding transcriptomes were enriched for replication, repair and cell-cycle signatures, whereas the opposite pairings showed lower bacterial burdens and stronger enrichment for endosomal, antigen-presentation and immune-signalling pathways in each cell type. Given the role of phospholipase C and virulence, we assessed *plc* expression and performed pharmacological inhibition experiments. These analyses revealed lineage- and macrophage-context-dependent expression patterns and reduced intracellular burden and cytotoxicity in THP-1m but not in BoMac. This framework links MTBC strain diversity to macrophage infection phenotypes and prioritizes candidate pathways potentially involved in bacterial–host interactions.

**Impact Statement**

Host association within the *Mycobacterium tuberculosis* complex (MTBC) is a long-recognized but poorly operationalized concept. This study introduces an epidemiology-guided *in vitro* framework that treats host–strain compatibility as a measurable, hypothesis-generating parameter rather than a fixed species-level trait. By analysing infection phenotypes and host transcriptional responses separately within two biologically distinct macrophage infection models, this work avoids over-interpreting cross-species comparisons and provides a rigorous template for studying MTBC host association *in vitro*. The framework integrates phenotypic, transcriptomic and functional perturbation data to prioritize candidate pathways for further validation in more physiological systems. More broadly, this study offers a practical experimental and analytical approach for linking MTBC strain diversity to host-context-dependent infection outcomes, with implications for understanding host range, adaptation and evolution in pathogenic bacteria.

## Data Summary

All data supporting the findings of this study are publicly available. RNA-seq data generated in this study have been deposited in the NCBI Sequence Read Archive under BioProject accession numbers PRJNA1124259 and PRJNA1124539. All specific accession numbers and processed data, including infection ratios, intracellular bacterial burden (c.f.u.) measurements, cell-death and cytotoxicity readouts, differential expression results, orthologue mappings and enrichment analyses, are provided in the Figshare repository from Microbiology Society associated with this article. Custom scripts used for RNA-seq processing, differential expression analysis, orthologue mapping and downstream visualization are available via the PathoGenOmics-Lab GitHub repository (https://github.com/PathoGenOmics-Lab/CrossInfectMTB). Figshare can be found here: https://doi.org/10.6084/m9.figshare.32983586 [1].

## Introduction

Host–pathogen specificity, the phenomenon by which pathogens infect certain hosts more successfully than others, is a cornerstone of understanding the ecology and evolution of infectious diseases. Such patterns can emerge when a pathogen exploits molecular or cellular mechanisms that enable more efficient entry, evasion of immune responses or replication within a given host [2, 3]. Beyond its importance to basic research, the study of pathogen specificity has crucial ramifications: it can inform vaccine design, guide targeted surveillance strategies and highlight potential avenues for zoonotic spillover, reinforcing the urgency of One Health approaches.

There are multiple examples of host range variation that illustrate how even subtle genetic changes can alter the host spectrum of a pathogen. In influenza A, small genomic shifts that modify receptor-binding affinities can drive outbreaks in new populations [4]. Similarly, coronaviruses can rely on discrete mutations to cross species barriers [5, 6]. However, in other pathogens, such as members of the *Brucellaceae* family, where different species infect different hosts, pinpointing the causal genetic changes is challenging, presumably because host adaptations can be ancestral and polygenic [7]. Members of the *Mycobacterium tuberculosis* complex (MTBC) also show non-random host associations across mammals [8], and identifying context-dependent bacterial factors that contribute to differential outcomes across host settings may ultimately inform host-directed intervention strategies.

*M. tuberculosis* is a major cause of infectious mortality worldwide and includes lineages that circulate predominantly in humans as well as lineages associated with animal hosts [8, 9]. While at least ten distinct phylogenetic lineages (L1–L10) circulate primarily among humans, there are also four animal-associated lineages (A1–A4) [10–13]. Lineage A1, including the chimpanzee bacillus among others, infects wild terrestrial animals in Africa and is notably understudied, despite being closely related to the human-associated Lineage 6 [14]. Although lineages display strong host associations, their tropism is not absolute [15]. For instance, Lineage A4, which includes *Mycobacterium bovis*, is predominantly found in cattle but can also persist in wildlife reservoirs such as deer, possums, badgers and wild boar, depending on the geographic setting, and is only occasionally transmitted to humans, with sustained human-to-human transmission being uncommon [16, 17]. Conversely, human-associated lineages can infect cattle and other animals, often with attenuated virulence and extremely rare spillback to humans [10, 14, 18, 19].

Notably, all non-human associated lineages (A1–A4) cluster within a clade that also includes the human-associated Lineages 5, 6, 9 and 10, collectively known as *Mycobacterium africanum*, although L5 branches earlier and differs substantially. Several lineages within this clade, particularly A1 and other animal-associated lineages, remain less well characterized than globally distributed human-associated MTBC lineages. Available data are comparatively stronger for L5, L6 and A4, but their phenotypic behaviour across controlled host-cell infection contexts remains incompletely resolved. L5 has been associated with Ewe ethnicity in Ghana [20], whereas L6 is phylogenetically closer to the animal-associated lineages, is more diverse than L5 and has been significantly associated with HIV co-infection in Ghanaian TB patients [21, 22]. These observations have led to hypotheses of differences in host association, with L6 potentially displaying a more generalist ecological profile compared to L5 [20]. Meanwhile, the host range and biology of other animal-associated lineages, especially the chimpanzee bacillus [23], which has been isolated only once despite being closely related to L6, remain poorly characterized, underscoring the need for additional experimental frameworks to prioritize testable hypotheses.

The molecular basis of differential outcomes across strains and hosts is an area of active research. Phospholipase C (*plc*) genes are plausible bacterial host determinants because they are preferentially deleted in animal-associated lineages [10, 24] and its activity is linked to macrophage cytotoxicity, necrotic cell death and altered eicosanoid signalling in *M. tuberculosis* infection models [25]. However, evidence for a general role of *plc* genes in MTBC virulence is inconsistent [26], and *plcD* was not identified as required for *M. bovis* survival in a bovine Tn-seq infection model [27]. In other bacteria, *plc* genes can aid immune evasion and membrane disruption [28, 29]. In the MTBC, *plc* activity has been linked to changes in eicosanoid signalling and to a bias towards necrotic outcomes over apoptosis [25]. Nevertheless, current evidence indicates that *plc* effects are neither universal nor sufficient on their own to explain cross-host outcomes [25, 30].

Despite progress in identifying genetic and molecular correlates of MTBC host range, substantial gaps remain in our ability to experimentally link long-term adaptation to particular hosts with measurable infection phenotypes and cellular response programmes under controlled conditions. It remains unclear whether phylogenetic affiliation reliably predicts *in vitro* infectivity and intracellular survival within a defined cellular context, and which molecular pathways most consistently track these outcomes across divergent MTBC lineages. Although previous work has examined MTBC infection in human, bovine, murine and other mammalian macrophage models, including comparisons involving *M. tuberculosis* and *M. bovis* [31–35], these studies have rarely combined MTBC strain diversity, infection phenotyping and host transcriptional profiling within the same controlled experimental framework. Consequently, parallel comparisons of several human- and animal-associated MTBC lineages, combining infectivity, intracellular bacterial burden and host transcriptional responses within the same experimental framework, remain limited.

Here, we establish a host-dependent *in vitro* experimental framework to operationalize host–strain compatibility as a theoretical parameter relevant to MTBC evolution. The framework is motivated by epidemiological observations stated above. First, because animal-associated lineages such as *M. bovis* (A4) circulate primarily in cattle and wildlife maintenance hosts, whereas human infections are generally zoonotic and are not typically associated with onward human-to-human transmission. We hypothesized that the highest compatibility for *M. bovis* would be bovine macrophages, whereas other closely related lineages, such as the chimpanzee bacillus and Lineage 6, would have lower compatibility. On the other extreme, human lineages outside the animal-associated clade would represent the least compatible combination, with Lineage 5 being the closest phylogenetically. On the other hand, human macrophages would show the opposite compatibility pattern, with highest compatibility with Lineage 5 and the lowest with *M. bovis*. First, we will evaluate these compatibility categories defined *a priori* based on epidemiological host association, using infection ratio, intracellular bacterial burden dynamics and cell death within each macrophage model. Then, we will profile host transcriptional responses by performing differential expression analyses separately within each model. Cross-model synthesis is restricted to one-to-one orthologue overlap and pathway-level enrichment summaries to highlight recurring macrophage response modules, without implying controlled species-level effects. Finally, as a proof-of-principle functional axis within this framework, we focus on the bacterial phospholipase C (*plc*) loci, which have been proposed as context-dependent contributors to virulence and host association in the MTBC. This analysis was included as an independent bacterial candidate axis, rather than as a pathway derived from the L5 vs *M. bovis* host transcriptomic contrast. We assess *plc* expression patterns across lineages and host contexts and explore whether pharmacological perturbation of *plc*-associated activity yields host-context-dependent effects on intracellular burden and cytotoxicity. In this way, the framework is intended to generate testable hypotheses and prioritize candidate mechanisms for validation in more physiological systems.

## Methods

### Cell lines and *M. tuberculosis* strains

Human THP-1-derived macrophages (THP-1m) and bovine macrophage cell line (BoMac) were used as two macrophage infection models. BoMac cells are an immortalized bovine peritoneal macrophage cell line generated by SV40 plasmid DNA transfection of primary bovine peritoneal macrophages [36]. THP-1 monocytes were maintained in Roswell Park Memorial Institute (RPMI) 1640 (Gibco, 21875034) supplemented with 10% FBS at 37 °C in a humidified incubator with 5% CO_2_. For differentiation, THP-1 cells were seeded in tissue-culture-treated 24-well or 6-well plates and treated with phorbol 12-myristate 13-acetate (PMA; 15 ng ml^−1^) for 24 h to generate THP-1 macrophage-like cells (THP-1m). After differentiation, the PMA-containing medium was replaced with fresh complete RPMI and cells were rested for 24 h at 37 °C with 5% CO_2_ prior to infection to allow stabilization and adherence.

BoMac cells were maintained at 37 °C with 5% CO_2_ in Iscove’s Modified Dulbecco’s Medium (IMDM) containing GlutaMAX and HEPES (Gibco, 31980–022) supplemented with 10% FBS, 1× MEM non-essential amino acids (Gibco, 11140–035), 1× MEM vitamins (Biochrom AG, K0373) and 50 µM β-mercaptoethanol (Gibco, 31350–010). For seeding, BoMac cells were detached using 0.25% trypsin/EDTA (Gibco, 25200–056). Cells were allowed to adhere and recover for 24 h at 37 °C with 5% CO_2_ before infection.

**Fig. 1.**
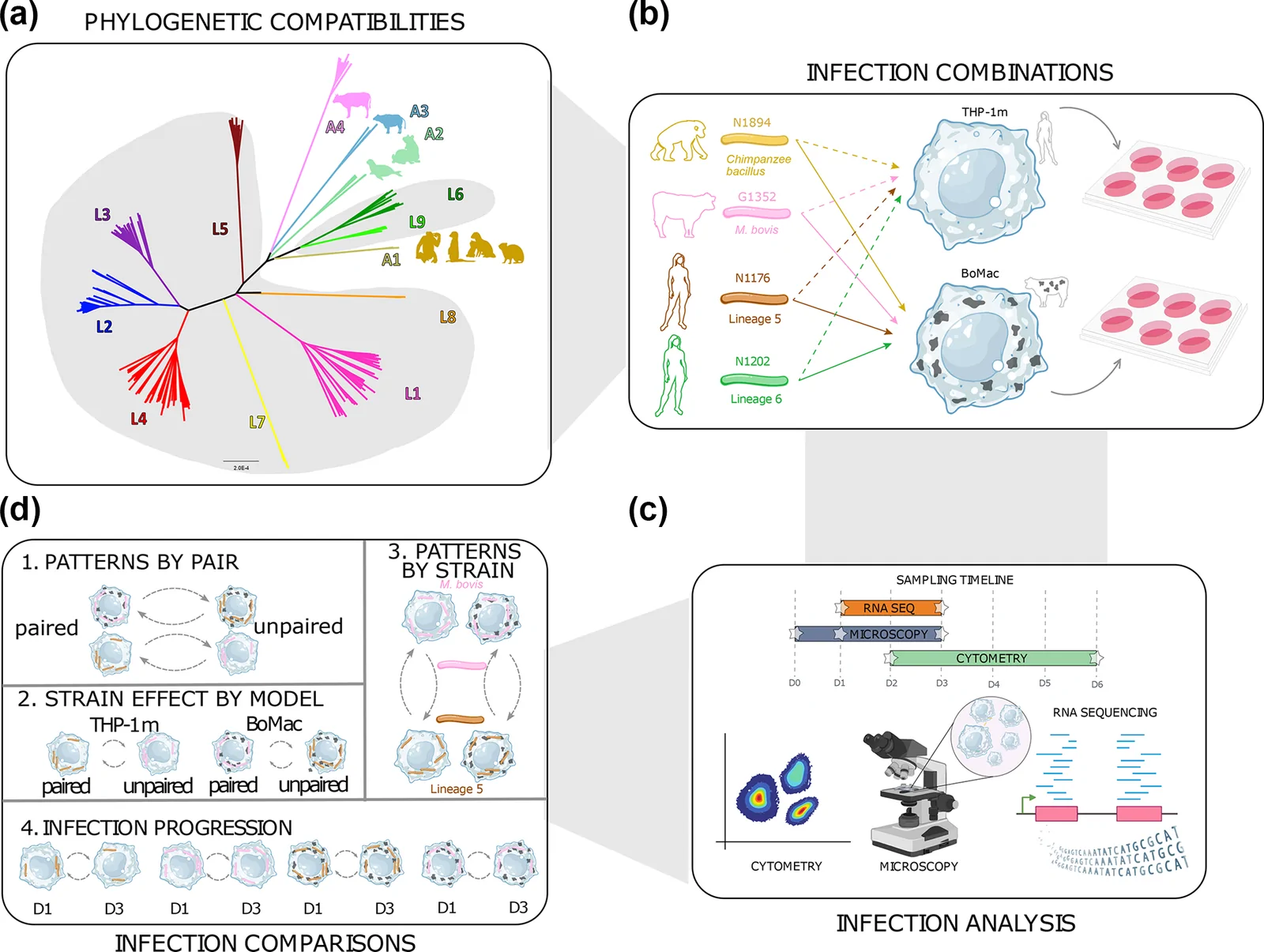
General experimental setup of crossed infections in *M. tuberculosis*. (**a**) Phylogenetic relationships among 14 *M*. *tuberculosis* lineages, with animal-associated lineages labelled with ‘A’ and a silhouette of their most common hosts, and human host-associated lineages with ‘L’ and shaded in grey. (**b**) Schematic representation of the crossed-infection experiments using four *M. tuberculosis* strains on THP-1m and BoMac cell lines. (**c**) Timeline of infection setup times and corresponding analysis techniques, including cytometry, microscopy, RNA extraction and sequencing. (**d**) Transcriptional comparisons for all infection combinations (strain–cell). For comparisons 1–3, analyses were conducted by concatenating and splitting data for 1 and 3 days post-infection (dpi).

Infections were made using a panel of four MTBC strains selected to represent contrasting host associations and phylogenetic positions within the complex, enabling comparisons between phylogenetically related strains with different host associations. Two human-associated strains were included: N1176 (Lineage 5, West African lineage, historically classified as *M. africanum*) and N1202 (Lineage 6, also human-associated but phylogenetically closer to animal-associated MTBC clades than Lineage 5) described in [37]. G1352 was included as a *M. bovis* strain from bovine-associated clade A4 [38], and N1894 was included as the chimpanzee bacillus, a primate-associated strain from phylogenetic clade A1, isolated from a wild chimpanzee and phylogenetically close to Lineage 6 [23] (strain details in Fig. 1a, b). Together, these strains capture both human- and animal-associated MTBC diversity, allowing us to explore the contribution of host association and phylogenetic relatedness to infection outcomes. All work with live mycobacteria was conducted in a biosafety level 3 facility following institutional biosafety procedures.

### *M. tuberculosis* infections

All strains were grown at 37 °C to mid-log phase (OD_600_ = 0.5–0.6) in Middlebrook 7H9 broth supplemented with 10% (v/v) albumin/dextrose/catalase (Middlebrook Enrichment, BD 212352), 0.44% (wt/v) sodium pyruvate and 0.05% (v/v) Tween-20. Bacterial cultures were washed in PBS and resuspended in the appropriate infection medium (RPMI for THP-1m; IMDM for BoMac). To generate single-cell suspensions and reduce clumping, suspensions were sonicated in a water bath sonicator at maximum power for 2 min.

Given that BoMac cells have been reported to show reduced phagocytic capacity compared with primary bovine mononuclear phagocytes [39, 40], infection conditions were optimized separately for each macrophage model. Preliminary optimization experiments using *M. bovis* BCG Pasteur confirmed lower bacterial uptake in BoMac cells than in THP-1m macrophages. Therefore, a higher m.o.i. was used for BoMac cells to partially compensate for this reduced uptake. THP-1m cells were infected at m.o.i. 10 and BoMac cells at m.o.i. 20 to obtain a measurable early infection window suitable for downstream c.f.u. enumeration, flow-cytometry and transcriptomic analyses. THP-1m and BoMac macrophages were seeded in six-well plates at 7×10^5^ and 5×10^5^ cells per well, respectively, and in 24-well plates at 5×10^5^ (THP-1m) and 1.8×10^5^ (BoMac) cells per well. Cells were infected with each strain–model combination at the indicated m.o.i. or left uninfected as controls.

At 4 hour post-infection (hpi), extracellular bacteria were removed and culture medium was replaced with RPMI or IMDM supplemented with gentamicin (5 µg ml^−1^). At 1 day post-infection (dpi), gentamicin-containing medium was replaced with fresh RPMI or IMDM supplemented with 10% FBS. Infected cultures were maintained for up to 6 days. Infections were performed in replicate wells for each strain–model combination (26 infections in total).

### *plc* Inhibition assay during infection

To evaluate the role of bacterial *plc*-related activity during infection, we used the *plc* inhibitors U73122 and D609 [25]. Single-cell suspensions of strains L5 and *M. bovis* were incubated with 10 µM U73122 (phosphatidylinositol-*plc* inhibitor; Sigma-Aldrich 662035) or 50 µM D609 (phosphatidylcholine-*plc* inhibitor; Sigma-Aldrich 251400) for 1 h at 37 °C with agitation. Suspensions were then centrifuged at 3,500 r.p.m for 10 min and washed twice with RPMI or IMDM prior to infection of THP-1m or BoMac cultures, respectively. Viability of treated inocula was assessed by c.f.u. enumeration to confirm that bacterial viability was not reduced by inhibitor exposure (Table S1, available in the online Supplementary Material).

Cell death in *plc* inhibition experiments was quantified by measuring lactate dehydrogenase (LDH) release using the CyQUANT™ LDH cytotoxicity assay (Invitrogen, C20301) according to the manufacturer’s instructions. At 4 hpi and at 1 and 3 dpi, culture supernatants were collected and 50 µl of supernatant was mixed with 50 µl of substrate mix, incubated for 30 min at room temperature in the dark and the reaction was stopped by adding 50 µl of stop solution per well. Absorbance was measured immediately using a Tecan microplate reader (Table S2).

### C.f.u. assays

At 4 hpi and at 1 and 3 dpi, cell culture medium was removed and infected monolayers were lysed by adding 100 µl of 0.1% Triton X-100 (Sigma-Aldrich) in water directly onto the cells followed by incubation for 20 min at 37 °C and 5% CO_2_. Macrophage lysates were serially diluted in PBS containing 0.05% Tween-80 and plated onto Middlebrook 7H11 agar supplemented with oleic acid–albumin–dextrose–catalase (OADC). Plates were incubated at 37 °C and 5% CO_2_, and colony growth was monitored for up to 6 weeks.

### Infection ratio by microscopy

Optical microscopy was performed on infected cells in 24-well plates (seeded at 5×10^5^ THP-1m cells or 1.8×10^5^ BoMac cells per well). The infection ratio was determined at 4 hpi and at 1 and 3 dpi (Fig. 1c). Cells were fixed with 3.7% formaldehyde and stained using the Ziehl–Neelsen method. Briefly, cells were stained with carbol fuchsin (Sigma-Aldrich, 108512), decolourized with hydrochloric acid in ethanol (Sigma-Aldrich, 100327) and counterstained with methylene blue (Sigma-Aldrich, 101287). Acid-fast bacilli retained the pink carbol-fuchsin staining, whereas decolourized macrophages were counterstained blue.

A macrophage containing at least one bacillus was classified as infected. Infected and uninfected macrophages were counted within a 44 mm^2^ area, and the infection ratio was calculated as the number of infected cells divided by the total number of cells in the observed area. Data were obtained across 12 experiments with at least two technical replicates for each infection combination (Table S3).

### Cell death assessment by flow cytometry

To assess macrophage cell death induced by infection, flow cytometry was performed at 2 and 6 dpi using infected six-well plates seeded with 7×10^5^ THP-1m cells or 5×10^5^ BoMac cells per well (Fig. 1c). Culture medium was removed, cells were detached using trypsin, washed with PBS and resuspended in 500 µl PBS.

Cells were stained following the Fixable Viability Stain 450 kit manufacturer’s instructions (BD Biosciences, 562247). Briefly, infected and uninfected macrophages were incubated with Fixable Viability Stain 450 diluted 1 : 10 in PBS for 5 min at 37 °C in the dark and then washed with PBS. Samples were subsequently stained with 80 µl CellEvent™ Caspase-3/7 Green Detection Reagent (Invitrogen, C10423) at 2 µM in PBS supplemented with 5% FBS and incubated for 30 min at 37 °C. Stained cells were fixed by adding 80 µl of 7.4% formaldehyde for 10 min at room temperature. After centrifugation, supernatants were removed, cells were resuspended in 500 µl PBS and samples were acquired on a BD LSRFortessa on the same day. Flow citometry standard (FCS) files were exported and analysed using FlowJo™ software v10.9.

After obtaining cell viability percentages for each sample, control samples identified as outliers were excluded using the interquartile range (IQR) method. Mean control values were calculated for each experiment, and fold changes were computed by dividing each sample value by the corresponding control mean. Values below 1 for apoptosis and necrosis and values above 1 for live cells were excluded; if one category failed across all samples, that category was removed. Additional outliers in fold change were also removed using the IQR method. After filtering, data from 12 experimental setups with at least two technical replicates per infection combination were retained for analysis (Table S4).

### RNA extraction and sequencing

RNA was extracted at 1 and 3 dpi (Fig. 1c) from infected macrophages cultured in six-well plates (7×10^5^ THP-1m cells or 5×10^5^ BoMac cells per well). Cells were lysed using RNAzol (500 µl per 5×10^5^ macrophages) and centrifuged for 20 min at 10,000 r.p.m. The supernatant was retained for eukaryotic RNA sequencing, whereas the pellet was processed for *M. tuberculosis* RNA extraction. Pellets from six wells were pooled, resuspended in 300 µl RNAzol and transferred to Lysing Matrix B tubes containing 0.1 mm silica beads (MP Biomedicals). Samples were vortexed for 3 min and centrifuged, and the resulting supernatants containing *M. tuberculosis* RNA were transferred to new tubes. Both bacterial and eukaryotic RNA fractions were then purified by 2-propanol precipitation followed by ethanol washes, and the final RNA was resuspended in 1 mM Tris/EDTA.

RNA-seq analyses included eight infection combinations with at least two technical replicates per infection combination and two uninfected controls with three technical replicates.

After extraction and purification, 48 samples underwent ribosomal RNA depletion using the Ribo-Zero Gold kit (Illumina, San Diego, USA). Library preparation was performed using the NEBNext^®^ SingleCell/Low Input cDNA Synthesis kit (New England Biolabs). Libraries were sequenced on an Illumina NovaSeq 6000 platform using an S4 150 bp configuration (see Table S5 for sequenced samples). Sequencing reads are available under BioProject IDs PRJNA1124259 and PRJNA1124539.

### RNA-seq analysis

Read quality was assessed, and trimming was performed using BBDuk from BBMap (v39.06). Reads were filtered by applying a minimum read length of 35 bp and a minimum quality threshold of Q35, retaining the longest high-quality sequences while discarding low-quality segments. Read quality was evaluated both before and after trimming using FastQC (v0.12.1).

Filtered reads were aligned using STAR (v2.7.11b) to the corresponding host reference genome for each macrophage model. Reads from infected THP-1m macrophages were mapped to *Homo sapiens* (Ensembl v104; accession GCA_000001405.15), whereas reads from infected BoMac macrophages were mapped to *Bos taurus* (Ensembl v109.12; accession GCA_002263795.2).

Downstream analyses were performed in R (v4.3.2); code is available at PathoGenOmics-Lab/CrossInfectMTB. Gene-level quantification was conducted using featureCounts from the R package (v2.16.1), assigning reads to genomic features using the Ensembl annotations matching each host genome release (Ensembl release 104 for *H. sapiens* and 109 for *B. taurus*). Raw count matrices were filtered using HTSfilter (v1.42.0) to remove genes with non-informative signal. Count normalization was performed using DESeq2 (v1.42.0) with the median-of-ratios method. Following exploratory analysis of gene expression profiles, one infected sample that clustered with uninfected controls was removed from downstream analyses.

### Differential expression analysis

Differential expression analyses were performed using DESeq2. Comparisons of interest are detailed in Fig. 1d. All differential expression contrasts were computed within each macrophage model separately (THP-1m and BoMac), and no direct THP-1m vs BoMac differential expression testing was performed. Cross-model synthesis was limited to one-to-one orthologue mapping and pathway-level enrichment summaries. Human and bovine gene identifiers were mapped using biomaRt (v2.58.0), retaining only one-to-one orthologues. Significant differentially expressed genes (DEGs) were defined using Benjamini–Hochberg adjusted *P*-value<0.05 and |log2 fold change|>1.

Gene Ontology (GO) enrichment analysis (GOEA) and Kyoto Encyclopedia of Genes and Genomes (KEGG) pathway enrichment analysis (KEA) were performed to identify enriched terms among DEGs using clusterProfiler (v4.10.0) and AnnotationDbi (v1.64.1). Host-specific annotation databases org.Hs.eg.db and org.Bt.eg.db (v3.19.1) were used for gene mapping during enrichment analyses. GO enrichment outputs were summarized and visualized using rrvgo (v1.14.1). Figures were generated using ggplot2 (v3.5.0).

### Differential exon usage analysis

Differential exon usage (DEU) was assessed after alignment by quantifying reads using the HTSeq Python library (v2.0.5). DEU analysis was performed using DEXSeq (v1.50.0). Alternative splicing analyses were executed using the nf-core/rnasplice pipeline (v1.0.3). Contrasts for comparisons of interest were evaluated using a custom R workflow, and DEU results were visualized using ggplot2 (v3.5.0) and DEXSeq outputs. Genes containing DEUs were subsequently subjected to GOEA and KEA using clusterProfiler (v4.10.0) and AnnotationDbi (v1.64.1).

### Reverse transcription quantitative PCR (RT-qPCR) assays of *plcABCD* genes

We quantified the expression of the four phospholipase C genes (*plcA*, *plcB*, *plcC* and *plcD*; see Table S6) by qPCR relative to *sigA*. For primer details, see Table S7.

In the retro transcription analysis, ~10 µl of RNA from *M. tuberculosis* derived from *in vitro* infections was denatured at 70 °C for 10 min. The RT reaction mix, consisting of 6 µl of 5× First Strand PCR buffer, 4 µl of 25 mM dNTPs, 6 µl of 25 mM MgCl_2_, 1 µl of 1 mg ml^−1^ random primers, 0.5 µl of SuperScript II reverse transcriptase and 22.5 µl of RNase-free water, was then added to the RNA. This mixture was incubated at 25 °C for 10 min, followed by 1 h at 42 °C for the reverse transcription.

For the qPCR reactions, the following components were used: 10 µl of SYBR Green, 2 µl of 10 µM primer mix, 6 µl of MilliQ H_2_O and 2 µl of template DNA/cDNA. The qPCR analysis was performed using a QuantStudio™ 3 Real-Time PCR System (Thermo Fisher Scientific) with the following protocol: (1) initial denaturation at 50 °C for 2 min and 95 °C for 10 min; (2) 40 cycles of annealing/extension at 95 °C for 15 s and with a ramp from 95 to 65 °C at 1.6 °C s^−1^, followed by 65 °C for 1 min; and (3) final dissociation at 95 °C for 15 s.

### *plcABCD* expression analysis

To assess the expression of the phospholipase C (*plc*) genes using qPCR, genomic DNA from a chimpanzee bacillus strain was used as a positive control at a concentration of 3×10^6^ genomes per qPCR reaction. The genome concentration was extrapolated based on the genome size of the H37Rv strain, following quantification of the genomic DNA extract using Qubit. For each case (combination of bacterial strain and macrophage type), three replicates were selected, focusing on those with the lowest Ct value for the *sigA* gene.

To calculate the relative expression of each gene, standard curves were generated using the genomic DNA of the chimpanzee bacillus as a template, since it contains the four phospholipase C genes. The number of copies in the qPCR reaction was inferred from the Qubit quantification and genome size. A logarithmic series of descending concentrations, starting from 3×10^6^ down to 0 copies, was created for each gene in each qPCR reaction.

A linear model was then fitted to the log_10_(template concentration), and the amplification efficiency (E) for each primer was determined by:

To determine the relative expression of each gene to *sigA*, we used the efficiencies of each primer and the Ct values, calculated by:

We normalized the variation of the relative expression given by replicates, where CtsigA_C_ is the Ct value of *sigA* amplification reaction of the positive control, with:

## Results

### Infection ratio and intracellular bacterial burden align with epidemiological pairing within each macrophage model

**Fig. 2.**
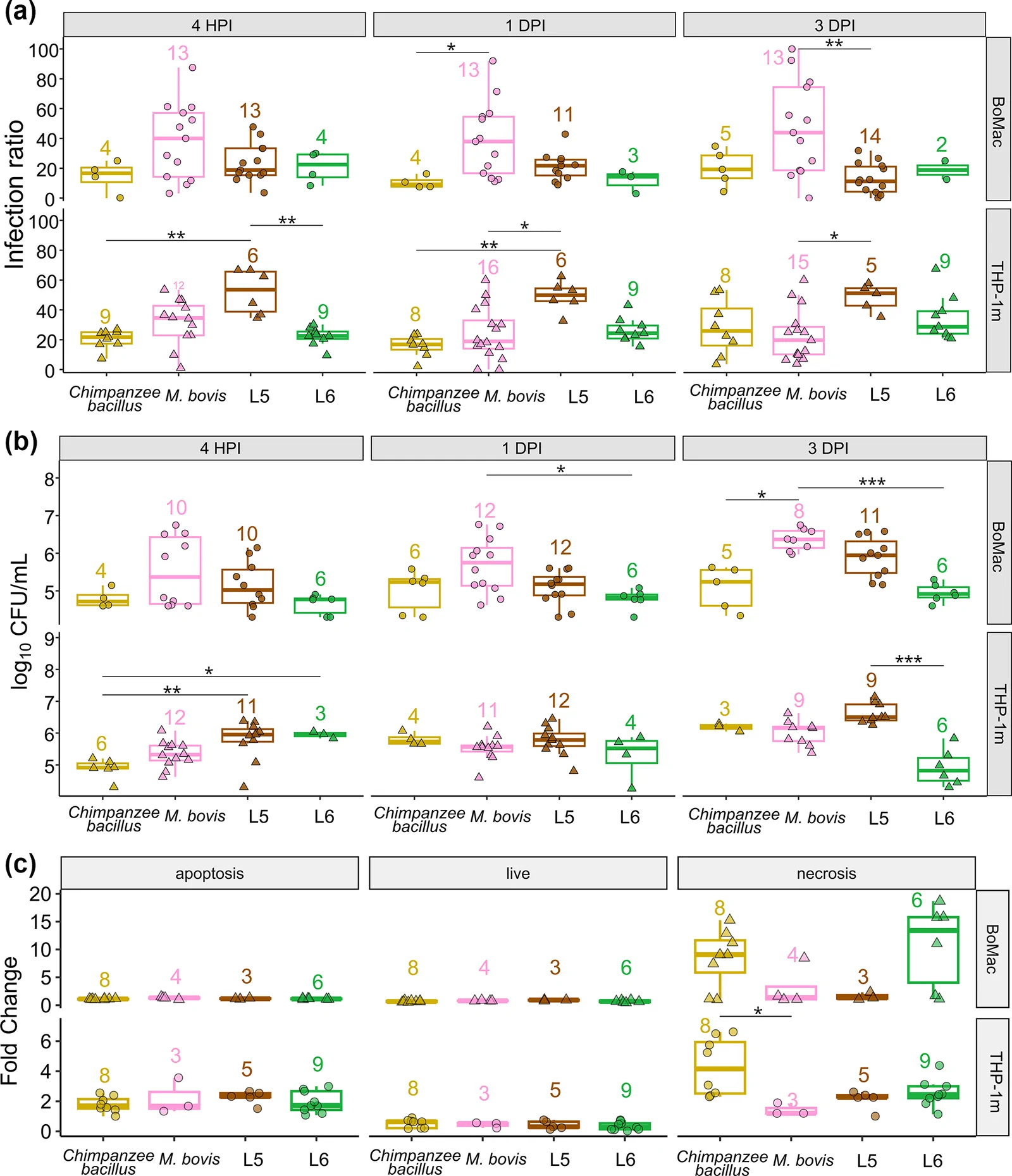
Strain-dependent infection phenotypes in THP-1m and BoMac macrophage infection models. (**a**) Infection ratio (percentage of infected macrophages) for four MTBC strains [chimpanzee bacillus, *M. bovis*, Lineage 5 (L5) and Lineage 6 (L6)] quantified at 4 hpi and at 1 and 3 dpi. (**b**) Intracellular bacterial burden measured by c.f.u. enumeration from infected macrophage lysates at the same time points, reported as log_10_ c.f.u. ml^−1^. For panels (a) and (b), BoMac data are shown in the upper row and THP-1m data in the lower row. (**c**) Infection-associated host cell death measured by flow cytometry on days 2 and 6 and reported as fold change relative to the uninfected control mean within each experiment for live, apoptotic and necrotic cells. Horizontal bars denote statistically significant pairwise comparisons; for clarity, only significant differences are shown (* *P_adj_*≤0.05, ** *P_adj_*≤0.01, *** *P_adj_*≤0.001).

To characterize strain-dependent infection phenotypes *in vitro*, we infected two widely used but biologically distinct macrophage models, human THP-1-derived macrophages (THP-1m) and bovine BoMac, with four MTBC strains (L5, L6, chimpanzee bacillus and *M. bovis*) and quantified infected-cell frequency at 4 hpi and at 1 dpi and 3 dpi (Table S3; Fig. 2a). Because THP-1m and BoMac differ in lineage and differentiation context beyond host species, we report strain effects within each model (Kruskal–Wallis followed by Dunn’s test with Bonferroni correction).

In THP-1m, infection ratios differed by strain at all time points (Kruskal–Wallis *P*=0.0011–0.0393), with the highest predicted compatibility strain, L5, consistently showing the highest infected-cell frequencies (median 49.8–53.5%). L5 exceeded A1 at 4 hpi (median 21.7%; *P_adj_*=0.00212) and 1 dpi (median 16.9%; *P_adj_*=0.00143), exceeded L6 at 4 hpi (median 22.6%; *P_adj_*=0.00640) and exceeded *M. bovis* at 1 dpi (median 18.9%; *P_adj_*=0.01339) and 3 dpi (median 19.7%; *P_adj_*=0.03391) (Table S3; Fig. 2a). In BoMac, infection ratios did not differ by strain at 4 hpi (*P*=0.3047), but strain-dependent differences emerged at 1 and 3 dpi (*P*=0.0132 and 0.0162), with the highest predicted compatibility strain, *M. bovis*, showing the highest infection ratios (median 37.9% at 1 dpi; 43.9% at 3 dpi) and exceeding A1 at 1 dpi (median 9.2%; *P_adj_*=0.01995) and L5 at 3 dpi (median 11.2%; *P_adj_*=0.00814) (Table S3; Fig. 2a).

We next quantified intracellular bacterial burden by c.f.u. at 4 hpi and at 1 and 3 dpi (Table S1; Fig. 2b). In THP-1m, overall strain effects were detected at 4 hpi (*P*=0.00377) and 3 dpi (*P*<0.001), but not at 1 dpi (*P*=0.174). At 4 hpi, both L5 (median 5.95 log_10_ c.f.u. ml^−1^) and L6 (median 5.96) exceeded A1 (median 4.91) (L5 vs A1: *P_adj_*=0.00855; L6 vs A1: *P_adj_*=0.04297), and by 3 dpi, the highest predicted compatibility strain, L5, remained high (median 6.50), whereas L6 was markedly lower (median 4.82; L5 vs L6: *P_adj_*<0.001) (Table S1; Fig. 2b). Although these differences did not reach statistical significance after Bonferroni correction, L5 was numerically higher than *M. bovis* at 4 hpi (5.95 vs 5.32 log_10_ c.f.u. ml^−1^; *P_adj_*=0.214) and showed a trend towards higher burden at 3 dpi (6.50 vs 6.16; *P_adj_*=0.0538). At 1 dpi, L5 and *M. bovis* burdens were similar (5.78 vs 5.56 log_10_ c.f.u. ml^−1^; *P_adj_*=0.456), consistent with the absence of an overall strain effect at this time point (Table S1; Fig. 2b).

In BoMac, strain effects were detected at 1 dpi (*P*=0.0398) and 3 dpi (*P*<0.001), but not at 4 hpi (*P*=0.286): the highest predicted compatibility strain, *M. bovis*, exceeded L6 at 1 dpi (5.75 vs 4.82 log10 c.f.u. ml^−1^; *P_adj_*=0.02903) and reached the highest burden at 3 dpi (median 6.36), exceeding A1 (median 5.24; *P_adj_*=0.01061) and L6 (median 4.92; *p_ad_* <0.001) (Table S1; Fig. 2b). *M. bovis* also showed numerically higher median c.f.u. than L5 at 1 dpi (5.75 vs 5.18; *P_adj_*=0.685) and 3 dpi (6.36 vs 5.94; *P_adj_*=0.640), although these contrasts were not significant after Bonferroni correction.

The higher c.f.u. counts in compatible strain–macrophage model combinations (Fig. 2b) may reflect variation in early bacterial uptake, intracellular recovery and/or differences in intrinsic replication rates. To distinguish this, we considered bacterial expansion normalized to the early post-infection burden for the two main epidemiology-informed reference strains: *M. bovis* and L5, calculated as the change in log_10_ c.f.u. between 4 hpi and 3 dpi (Table S8). This result did not support that highly compatible combinations resulted in higher bacterial expansion (Table S8). Therefore, the higher bacterial burden in this short timeline should be interpreted as differences in bacterial uptake and/or early intracellular recovery rather than just short-term intracellular expansion.

Finally, we assessed cytotoxic outcomes by flow cytometry at 2 and 6 dpi, quantifying normalized readouts for apoptosis, live-cell abundance and necrosis within each macrophage model (Table S4; Fig. 2c). To focus on within-model strain effects, we compared strains separately within THP-1m and within BoMac using Kruskal–Wallis with Dunn’s test with Bonferroni correction. Within THP-1m, there were no significant strain-associated differences in apoptosis (Kruskal–Wallis *P*=0.717) or live-cell abundance (*P*=0.625), despite modest variation in medians across strains (apoptosis medians 1.68–2.33; live-cell medians 0.27–0.64). In contrast, necrosis differed by strain at the global level (*P*=0.0158), driven by higher necrosis for the chimpanzee bacillus (median 4.16) than for *M. bovis* (median 1.20*; P_adj_*=0.0211).

Within BoMac, strain effects were not significant for apoptosis (*P*=0.193), live-cell abundance (*P*=0.121) or necrosis (*P*=0.0936). Notably, necrosis showed substantial dispersion, with higher medians for L6 (13.40) and the chimpanzee bacillus (9.07) than for L5 (1.30) and *M. bovis* (1.35). Together, these cytometry data indicate that strain-associated differences in cell-death readouts are modest within each macrophage model, supporting our focus on earlier infection and intracellular burden phenotypes and motivating subsequent within-model transcriptomic contrasts (Tables S1, S3 and S4; Fig. 2a–c).

### Strain-dependent host transcription in distinct macrophage infection models

**Fig. 3.**
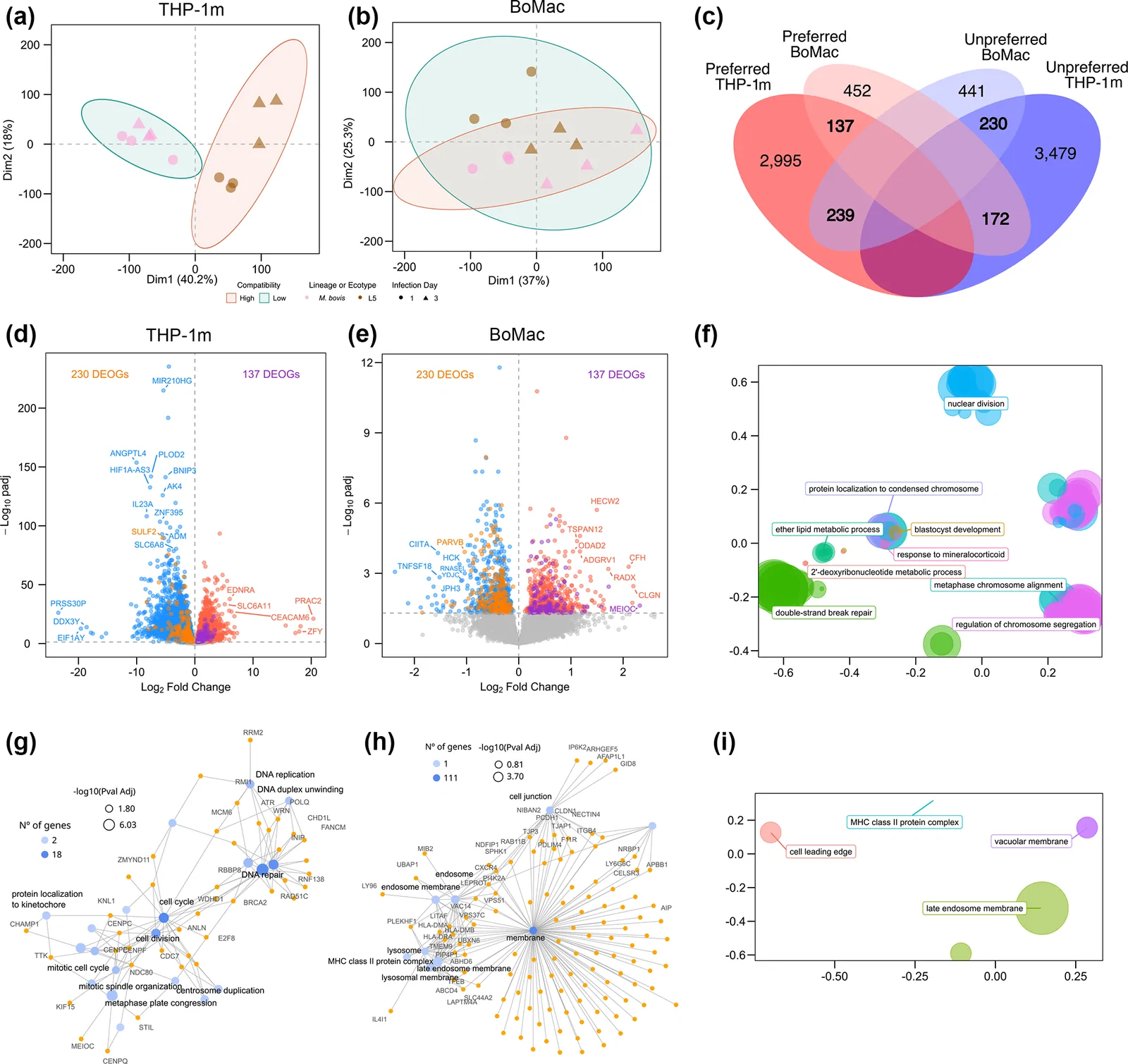
Strain-dependent host transcriptional responses in THP‑1m and BoMac macrophage infection models. (a, b) Dimensionality reduction of host RNA‑seq profiles across all infection conditions in THP‑1m (a) and BoMac (b) macrophages. (c) Overlap of significantly differentially expressed one‑to‑one orthologues (DEOGs) identified in the within‑model contrast between the epidemiologically preferred and unpreferred strain–model pairs (THP‑1m: L5 vs *M. bovis*; BoMac: *M. bovis* vs L5). (d, e) Volcano plots of DEG results for the preferred vs unpreferred within‑model contrasts in THP‑1m (d) and BoMac (e). Gene labels are shown only for genes meeting the significance threshold (adjusted *P*-value < 0.05) and a large effect-size cutoff (THP‑1m: |log2 fold change|>5; BoMac: |log2 fold change|>1). (f, g) GO biological process enrichment for the 137 DEOGs upregulated in the preferred‑pair direction. Panel (f) shows all GO term similarity structure, and panel (g) shows the top 20 enriched biological processes. (h, i) GO cellular component enrichment for the 230 DEOGs upregulated in the unpreferred‑pair direction. Panel (i) shows all GO terms, and panel (h) shows the top 10 enriched cellular components. In panels (f) and (i), distances between points reflect similarity among GO terms, and point size indicates the GO term score.

Global host transcriptome profiling revealed that infection induces strong, strain-dependent transcriptional responses in both macrophage models, with clearer separation among conditions in THP-1m (Fig. 3a, b).

Infections with L5 and *M. bovis* contributed most prominently to the major axes of variance in THP-1m(Fig. 3a), whereas BoMac showed a more compact global structure with strain-dependent separation still detectable(Fig. 3). Notably, the direction of these global patterns is concordant with the epidemiology-informed reference pairings defined *a priori* within this framework: L5 contributed most to transcriptomic separation in THP-1m and *M. bovis* contributed most in BoMac. Accordingly, we focused subsequent differential expression analyses on within-model contrasts between L5 and *M. bovis*, representing the two reference strains with opposite predicted compatibility across the human and bovine macrophage models. Importantly, because THP-1m and BoMac are biologically distinct macrophage model systems that differ in lineage and differentiation context beyond host species, we did not perform any direct THP-1m vs BoMac differential expression testing; instead, all contrasts were computed within each model, and any cross-system synthesis was limited to summarizing recurring orthologue- and pathway-level signals across the two model systems.

Within THP-1m, the L5 vs *M. bovis* comparison identified 2,456 DEGs increased in L5 infection and 3,654 DEGs increased in *M. bovis* infection (Table S9; Fig. 3c, d and S2A). Genes increased in L5 infection were enriched for pathways related to replication and repair, including cell cycle and DNA replication, together with additional metabolism-associated terms (Table S10). In contrast, genes increased in *M. bovis* infection in THP-1m showed strong enrichment for immune and signalling programmes (Fig. S2B), including production and regulation of signalling molecules, cellular communication and transduction, regulation of T-cell activation and cytokine/IL-related processes; additional enrichment included lipid metabolism and oxygen-related responses (Table S10). Within BoMac, the corresponding within-model contrast identified 758 DEGs increased in *M. bovis* infection and 910 DEGs increased in L5 infection (Table S9; Fig. 3e d).

In BoMac, genes increased in *M. bovis* infection were enriched for functions related to cell cycle, DNA replication, helicase and ATP-associated activities, whereas genes increased in L5 infection were enriched for antigen processing and presentation and T helper cell differentiation terms (Table S10). Together, these results indicate that the two macrophage models exhibit distinct strain-associated response landscapes and that the direction of the dominant programme differs by model: a replication/repair signature is prominent under one strain in each system, whereas immune/containment-associated programmes are prominent under the alternative strain (Tables S9 and 10; Fig. 3c-f).

To identify transcriptional features that recur across these two divergent macrophage systems, we used a conservative orthologue-mapping strategy. Specifically, after computing DEGs separately in THP-1m and BoMac, we intersected the within-model DEG lists to identify orthologues that were significantly regulated in both systems with concordant directionality relative to the local strain contrast (Table S11; Fig. 3g, i). This analysis identified 137 differentially expressed one-to-one orthologues (DEOGs) that were consistently increased in the strain associated with epidemiologically preferred pairs within each model (THP-1m: L5; BoMac: *M. bovis*) and 230 orthologues consistently increased in the direction of the unpreferred pairs (THP-1m: *M. bovis*; BoMac: L5; Table S11). Functional enrichment of the 137 shared orthologues highlighted cell cycle regulation and DNA replication, including nucleolus-associated terms, and KEGG enrichment included cell cycle and Fanconi anaemia pathways (Table S12; Fig. 3f and g). In contrast, the 230 shared orthologues were enriched for terms consistent with intracellular bacterial containment and immune-related processes, including late endosome/vacuolar membrane and MHC class II-associated components (Table S12; Fig. 3h, i and S3). Among these genes, we identified *SLC7A7*, previously linked to nitric oxide-related antimicrobial responses in macrophages [41], consistent with a containment-oriented module being engaged under these conditions.

Finally, we aimed to identify infection-induced host responses that were attenuated under conditions associated with higher intracellular burden. We therefore selected genes that were upregulated in at least one infected condition compared with uninfected controls, but were expressed at lower levels in the higher-burden strain than in the lower-burden strain within each macrophage model. This approach identified 533 genes in BoMac, 3,188 genes in THP-1m and 95 one-to-one orthologues shared across both systems (Table S13). In BoMac, these candidates were enriched for Golgi vesicle transport and peroxisomal membrane transport, implicating vesicle trafficking and peroxisomal functions in bovine macrophage responses (Table S14). In THP-1m, enriched pathways included cytokine–cytokine receptor interaction, TNF, NFκB, MAPK, chemokine, ferroptosis, IL-17 signalling, B cell receptor signalling, Th1/Th2 differentiation and peroxisomal transport/protein import, together with intrinsic apoptotic signalling, positive regulation of kinase activity, glycolysis and response to hypoxia (Table S14; Fig. S2C).

To contextualize these attenuated-response candidates within the broader inflammatory programme, we next interrogated a curated 374-gene inflammatory/innate-immune panel. Consistent with canonical macrophage activation, infection induced a robust pro-inflammatory programme in THP-1m cells. Compared with uninfected controls, 126 of the 340 detected genes in this panel were significantly upregulated, using a Benjamini–Hochberg-adjusted threshold of *P_adj_*<0.05. These included canonical cytokines (TNF, IL6, IL1A, IL23A), chemokines (CXCL8, CXCL9, CXCL10) and effector genes (PTGS2/COX2, IDO1), with log_2_ fold changes of ~1.6–6.7 and the strongest responses (Fig. S1). IL1B was induced in a strain- and time-dependent manner, reaching significance during *M. bovis* infection, whereas NOS2 was not induced, consistent with expectations for human macrophages. In BoMac cells, this inflammatory panel was less informative: the canonical NFκB-associated cytokines TNF, IL6 and IL1B were not detected in either controls or infected, while only the IFN-driven node, including CXCL10, was clearly induced (Fig. S1).

Collectively, these results show that strain-associated transcriptional programmes are largely model-specific, while a restricted set of orthologue-level signatures – reflecting broad macrophage modules such as replication/repair vs endosomal/antigen-presentation/immune processes – can be detected as recurring patterns across two biologically distinct macrophage infection systems (Tables S9–S14; Fig. 3).

### Strain-specific transcriptional signatures shared across macrophage models

Beyond the within-model strain contrasts described above, we asked whether any host transcriptional features were consistently associated with a given strain across both macrophage infection systems, in two independent macrophage models. To do so, we intersected the one-to-one orthologue results obtained independently from the THP-1m and BoMac L5 vs *M. bovis* contrasts and retained orthologues showing concordant directionality in both models (Table S11; Fig. S4).

This orthologue-level synthesis identified 239 DEOGs that were consistently upregulated during L5 infection relative to *M. bovis* (Table S11). Functional enrichment of these L5-associated genes highlighted processes related to peroxisomes/microbodies, vesicle tethering complexes and chromosomal or telomeric regions (Table S12). Conversely, 170 DEOGs were consistently upregulated in *M. bovis* infection relative to L5 (Table S11). These *M. bovis*-associated genes were enriched for ferroptosis-related pathways and included molecular functions such as ubiquitin-like protein ligase activity and cadherin binding, with enrichment for cellular components including the rough endoplasmic reticulum membrane (Table S12).

### Temporal dynamics of strain-associated transcriptional expression

To examine how host transcriptional responses evolve over the course of infection, we performed time-stratified, within-model differential expression analyses at 1 and 3 dpi (THP-1m: L5 vs *M. bovis*; BoMac: *M. bovis* vs L5; Fig. S5) and then used one-to-one orthologue mapping to summarize recurring, time-dependent signatures across the two macrophage infection systems (Table S15; Fig. S5). This approach avoids any direct THP-1m vs BoMac differential expression testing and instead identifies orthologues that change in the same direction in both models relative to the local strain contrast.

At 1 dpi, the orthologue-level synthesis identified 528 DEOGs consistently increased in epidemiological preferred pairs within each model (THP-1m: L5; BoMac: *M. bovis*) (Tables S10–S11). These genes were enriched for KEGG pathways linked to signal transduction, infectious disease, focal adhesion and metabolism of terpenoids and polyketides (Table S16; Fig. S5). GO enrichment highlighted ribosome biogenesis, RNA processing and regulatory processes including negative regulation of apoptotic signalling (Table S16; Fig. S5). In the reciprocal direction at 1 dpi, 367 DEOGs were consistently increased (Tables S10–S11) and showed enrichment for pathways including pentose phosphate, thermogenesis and oxidative phosphorylation (Table S16). Corresponding GO terms included ribose phosphate biosynthetic process, energy derivation by oxidation of organic compounds, response to oxygen levels/oxidative stress, regulation of leucocyte apoptotic process and vesicle-mediated transport between endosomal compartments (Table S16; Fig. S5).

At 3 dpi, the orthologue-level synthesis identified 638 DEOGs consistently increased in the higher-phenotype strain direction (Table S15; Fig. S5), with enrichment for lysosomal pathways, phosphatidylinositol signalling, inositol-phosphate metabolism, DNA replication and glycan degradation (Table S16; Fig. S5). In the reciprocal direction at 3 dpi, 266 DEOGs were consistently increased (Table S15; Fig. S5) and were enriched for molecular functions related to actin binding, energy-dependent membrane transport, NAD(P)H-driven electron transfer (including NADH/NAD(P)H dehydrogenases), small-GTPase and MAP-kinase signalling, NAD binding and carbohydrate kinase activity (Table S16).

### Differential exon usage in THP-1m macrophages depends on the infecting strain

**Fig. 4.**
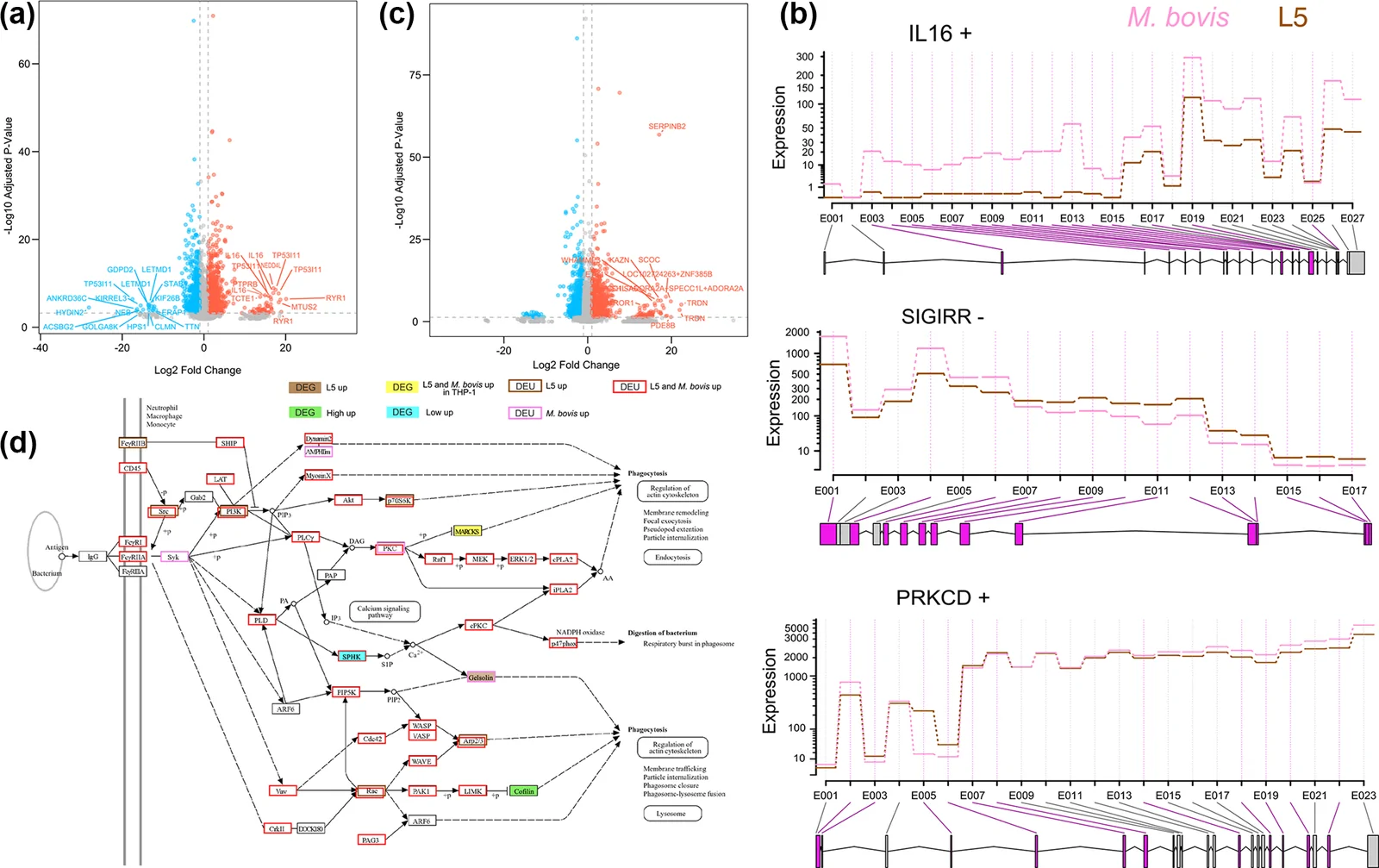
Strain-associated DEU in THP-1m macrophages. (**a**) DEU for genes showing exon-specific changes between infections. Each dot represents an exon; blue indicates exons with higher usage in L5-infected THP-1m cells and red indicates exons with higher usage in *M. bovis*-infected THP-1m cells within the same gene. (**b**) Fitted exon-level expression profiles for *IL16*, *SIGIRR* and *PRKCD*. Exons with significant differential usage between L5 and *M. bovis* infections are highlighted in purple. (**c**) Genes showing concordant exon-wide shifts between infections. Dots represent exons from genes for which all exons are more highly expressed in L5 infection (blue) or all exons are more highly expressed in *M. bovis* infection (red). (**d**) KEGG Fc gamma R-mediated phagocytosis pathway annotated with DEGs and DEUs. Genes identified as DEGs are shown as filled boxes, and genes with significant DEUs are shown as outlined boxes. The direction of change for each contrast is indicated in the legend.

In THP-1m macrophages, infection with L5 or *M. bovis* was accompanied by widespread DEU, indicating that isoform-level regulation may contribute to the host response. Using exon-level testing, we identified 982 genes with significant DEU between L5- and *M. bovis*-infected THP-1m macrophages (*P_adj_*<0.05 and |log_2_ fold change|>1; Fig. 4a, Table S17). These genes were enriched for functions related to cell growth, cellular organization and motility, together with GTPase activity (Table S18), consistent with strain-associated isoform regulation affecting cytoskeletal dynamics, signalling and migration. Enriched KEGG pathways included extracellular matrix–receptor (ECM) interaction, focal adhesion and cytoskeleton organization (Table S18), suggesting that strain-dependent exon usage may contribute to structural adaptations of macrophages during infection.

At the single-gene level, *IL-16* (28 exons) showed 15 DEU events (*P_adj_*<0.05), with exons E025 and E019 upregulated in *M. bovis* infection, while the remaining 13 DEUs were upregulated in L5 infection (Fig. 4b; Table S17). Given *IL-16*’s role in immune signalling and T-cell recruitment, these isoform differences could influence inflammatory responses. *SIGIRR* (17 exons), a negative regulator of Toll-like receptor and *IL-1* receptor signalling, displayed 14 DEU events, with 11 exons upregulated in L5 infection and 3 upregulated in *M. bovis* infection (Fig. 4b; Table S17). In addition, *PRKCD* (23 exons), a regulator of apoptosis with context-dependent effects on cell death, exhibited six DEU events, split evenly between L5- and *M. bovis*-associated exon usage (Fig. 4b; Table S17).

Beyond discrete exon-level events, we also identified genes showing consistent exon-wide shifts between strains. Specifically, 2,379 genes had all exons more highly expressed during L5 infection (*P_adj_*<0.05 and log_2_ fold change<−1; Fig. 4c; Table S17). These genes were enriched for biological processes including autophagy regulation, phospholipid metabolism, protein localization to the nucleus, G1/S transition, GTPase-mediated signal transduction and glucose-6-phosphate metabolism (Table S18). Enriched molecular functions included actin binding and DNA binding, while enriched cellular components were consistent with broad programmes related to cellular organization (Table S18). Conversely, 1,749 genes had all exons more strongly expressed during *M. bovis* infection (*P_adj_*<0.05 and log_2_ fold change>1; Fig. 4c; Table S17). These genes were enriched for processes such as regulation of endocytosis, peptidyl-serine phosphorylation and protein modification (Table S18), together with functions including GTPase regulator activity, nucleoside-triphosphatase regulator activity and ATP hydrolysis-related activities, consistent with signalling and energetic demands during infection. Enriched cellular components included the cleavage furrow, histone acetyltransferase complex, mitotic complex and nt-excision repair complex (Table S18). KEGG enrichment further included pathways related to DNA replication, Fc gamma R-mediated phagocytosis, biosynthesis of cofactors and VEGF signalling (Table S18).

Among the pathways showing notable DEU, Fc gamma R-mediated phagocytosis emerged as a prominent process with strain-associated isoform usage. *SPHK*, for example, was differentially spliced and upregulated during *M. bovis* infection in THP-1m (Fig. 4d), consistent with sphingosine-1-phosphate production and its role in actin dynamics and phagocytic efficiency [42]. In contrast, cofilin showed increased expression at the gene level in L5 infection (Fig. 4d), consistent with its role in actin remodelling and phagosome–lysosome fusion [43]. *MARCKS* remained upregulated in THP-1m regardless of strain (Fig. 4d), supporting a central role for this factor in membrane trafficking and cytoskeletal regulation during phagocytosis.

### Macrophage model shapes infection outcomes associated with phospholipase C

**Fig. 5.**
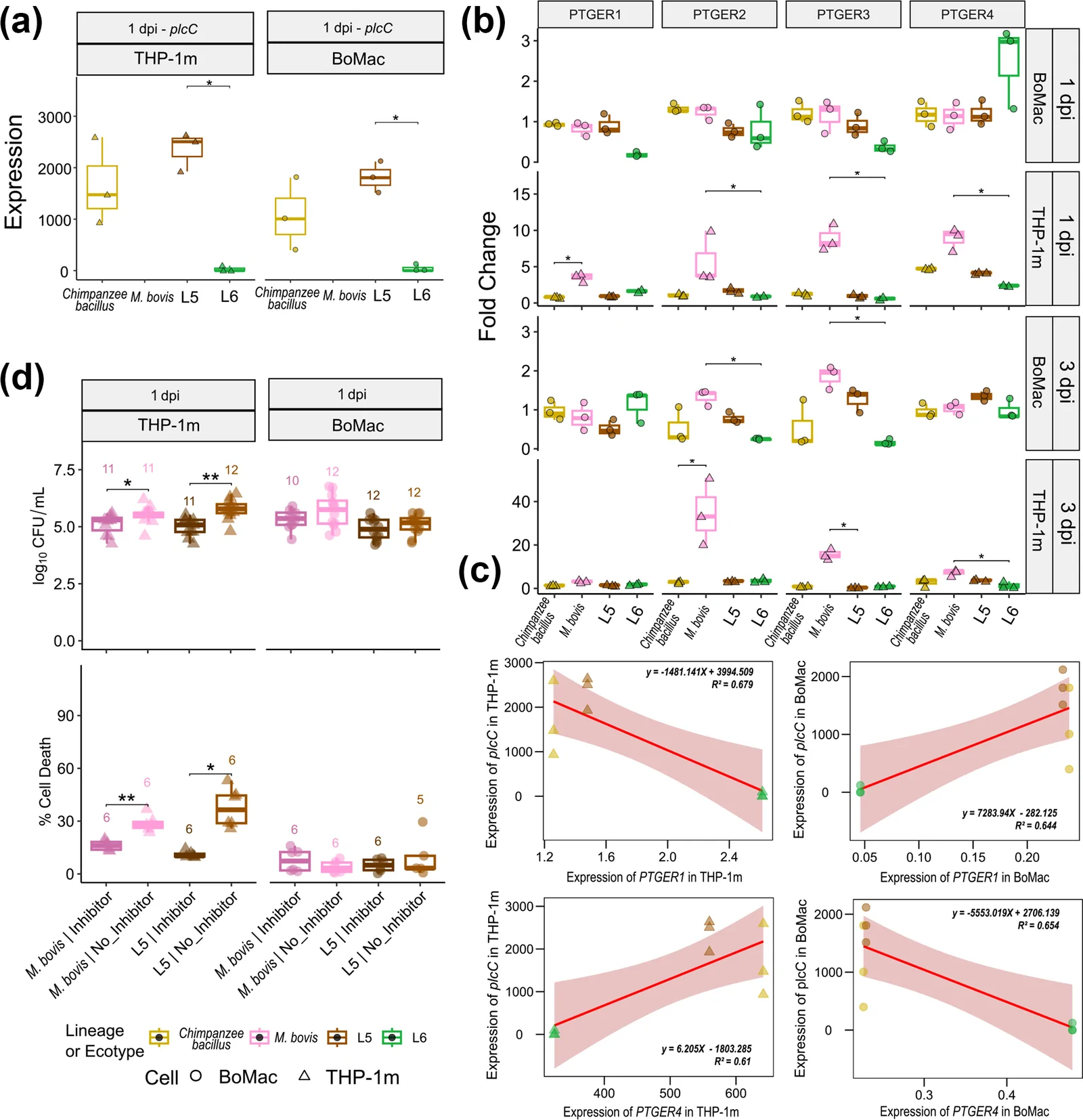
Lineage- and host-cell-context-dependent expression of mycobacterial phospholipase C genes and host *PTGER* receptors in THP-1m and BoMac infection models. (**a**) Relative expression of *plcC* at 1 dpi during infection of THP-1m and BoMac macrophage models with the chimpanzee bacillus, *M. bovis*, L5 and L6. (**b**) Host PTGER1–PTGER4 expression at 1 and 3 dpi in THP-1m and BoMac, expressed as fold change relative to time-matched uninfected controls (fold changes calculated per experiment using the control mean). (**c**) Correlation between bacterial *plcC* expression and host *PTGER*1 or *PTGER*4 expression in THP-1m and BoMac infections at 1 dpi. (**d**) Effect of *plc*-associated activity inhibition on infection outcomes at 1 dpi. Intracellular bacterial burden is shown in the upper panel and cytotoxicity, measured by LDH release, is shown in the lower panel, for THP-1m and BoMac infections under untreated and inhibitor-treated conditions. For panels (a–d), only significant comparisons are annotated.* *P_adj_*≤0.05; ** *P_adj_*≤0.01.

To explore host–pathogen crosstalk involving phospholipase C, we quantified mycobacterial *plc* transcript abundance across strains during infection of THP-1m and BoMac macrophage models at 1 and 3 dpi and, in parallel, measured host prostaglandin E receptors (*PTGER*1*–*4) expression under the same conditions (reported as fold change relative to host- and day-matched uninfected controls). We then tested whether pharmacological modulation of *plc*-related signalling during infection altered infection readouts in a different experiment (Fig. 5).

Mycobacterial *plc* transcription was strain- and time-dependent within both infection models (Fig. 5a). In THP-1m, *plcC* differed by strain at 1 dpi (Kruskal–Wallis *P*=0.0496), driven by undetectable *plcC* in L6 (median=0) relative to L5 (median=2,504.0; Dunn–Bonferroni *P_adj_*=0.0498). In BoMac, *plcC* also differed by strain at 1 dpi (Kruskal–Wallis *P*=0.0496), with *plcC* undetectable in L6 (median=0) and higher expression in L5 (median=1,805.2; Dunn–Bonferroni *P_adj_*=0.0498). In both THP-1m and BoMac, no significant strain-associated differences were detected for *plcA*, *plcB* or *plcD* at 1 dpi, and no significant strain-associated differences were detected across *plcA–D* at 3 dpi (*P*>0.05 in all panels). In *M. bovis*, *plcABC* expression was not detected because the RD5-associated deletion removes these genes from the genome.

Host *PTGER* responses also depended on infection context, strain and time (Fig. 5b). In THP-1m, fold changes in *PTGER*1–4 differed by strain at 1 dpi (Kruskal–Wallis *P*=0.0240–0.0370) and at 3 dpi (*P*=0.0237–0.0415), with day-1 post-hoc differences primarily involving *M. bovis* (Dunn–Bonferroni significant comparisons: *P_adj_*=0.0188–0.0494). In BoMac, no strain effects were detected at 1 dpi (*P*=0.109–0.316), and strain effects at 3 dpi were limited to PTGER2 (*P*=0.0378) and PTGER3 (*P*=0.0273; Fig. 5b).

When all strains were analysed together, pooled correlations suggested negative associations between PTGER1–4 and *plcA/plcB*, and between PTGER2/3 and *plcC* (Fig. 5). Given that *M. bovis* lacks the *plcA*, *plcB* and *plcC* loci while retaining *plcD*, and that L6 showed negligible *plcC* expression, we re-analysed the correlations after excluding values corresponding to genes absent from the *M. bovis* genome (Table S6). In THP-1m L5 infections, *plcC* correlated positively with *PTGER*1 (*P*=0.00630) and negatively with *PTGER*4 (*P*=0.0129); in BoMac L5 infections, *plcC* correlated negatively with *PTGER*1 (*P*=0.00925) and positively with *PTGER*4 (*P*=0.00832) (Fig. 5c).

We further explored the role of phospholipase activity by inhibiting bacterial PLC-associated activity with pharmacological inhibitors (U73122 and D609) prior to infection. We quantified intracellular bacterial burden and cytotoxicity (Fig. 5d; Table S2), and confirmed inoculum viability by c.f.u. enumeration (Table S1). In THP-1m, treatment with inhibitor reduced intracellular burden for L5 and *M. bovis* (L5 : 4.98 inhibited vs 5.76 untreated log_10_ c.f.u. ml^−1^, *P_adj_*=0.002; *M. bovis*: 5.11 vs 5.52, *P_adj_*=0.03) and reduced cytotoxicity (L5 : 10.96% vs 37.57%, *P_adj_*=0.03; *M. bovis*: 16.16% vs 28.60%, *P_adj_*=0.03; Mann–Whitney U with Benjamini–Hochberg correction). In BoMac, the same inhibitor treatment did not produce significant changes in intracellular burden (L5 : 4.88 vs 5.08 log_10_ c.f.u. ml^−1^; *M. bovis*: 5.31 vs 5.68) or cytotoxicity (L5 : 4.85% vs 9.33%; *M. bovis*: 7.70% vs 3.97%) (Fig. 5d; Table S2).

## Discussion

Epidemiological observations on *M. tuberculosis*–host associations can be interpreted as a theoretically ‘host compatibility’ concept, which can be experimentally tested by comparing measurable infection readouts in macrophage infection models. Here, we use two established *in vitro* macrophage cell lines: THP-1m and BoMac to examine how macrophage context and bacterial strain shape infection phenotypes and host transcriptional programmes, and we found that bacterial burden and host transcriptional response converge by predicted compatibility in each infection model. Because the cell lines used in this study differ in species of origin, cell source and differentiation history, direct comparison between cell lines would not accurately disentangle the role of host factors from cell line history. Instead, we address the study of host specificity by comparing the relative performance of different bacterial strains within each cell model when each cell line includes a strain predicted to be better paired to a host. In this case, the identification of consistent infection patterns across the different models can reveal host-specific signatures while minimizing the confounding effects of intrinsic differences between cell lines.

Across infection phenotyping assays, we observed polarized outcomes for two host-associated strains. Within the THP-1m model, L5 showed higher infection ratios than *M. bovis*; within the BoMac model, *M. bovis* showed higher infection ratios than L5. The reciprocal strain–model combinations produced the lowest infected-cell frequencies. These trends were mirrored by intracellular bacterial burden at later time points, with the strain that achieved higher infected-cell frequencies within a given model also tending to show higher or more sustained c.f.u. trajectories. In contrast, the chimpanzee bacillus and L6 displayed intermediate infection ratios and infected both macrophage models at more similar rates. Although L6 is generally associated with human infections, cases in immunocompromised individuals have been reported [20], and its phylogenetic placement among animal-associated lineages and increased genomic diversity support the view that L6 differs from L5 and can exhibit distinct infection behaviour *in vitro*. Differences in cell line origin could play a role in the infection ratio and intracellular bacterial burden observed. These could be attributed to differences in the lower expression of receptors, such as CR3 (CD11b/CD18) and CD14, which are involved in mycobacterial uptake and signalling [39, 40, 44]. Reduced expression of these receptors may affect both phagocytosis and early signalling responses to *Mycobacterium* strains in BoMac. However, our study does not rely on direct phenotypic comparisons between THP-1-derived macrophages and BoMac cells. Rather, comparisons were performed among bacterial strains within each cell model. Consequently, any intrinsic differences in receptor expression or cellular responsiveness are expected to similarly affect all strains within a given cell line and are therefore unlikely to account for the strain-specific infection patterns observed. The bacterial burden differences between compatibility pairs could be due to bacterial uptake differences, persistence and/or bacterial replication. The 72 h macrophage infection window used here is relatively short for slow-growing MTBC strains and limits our ability to quantify bacterial replication and therefore distinguish between the different mechanisms influencing bacterial burden. Longer and more physiological infection models may therefore be required to resolve host-associated differences in replication dynamics.

To explore molecular programmes linked to these phenotypic differences, we focused the transcriptomic analyses on the L5 and *M. bovis* contrasts within each macrophage model, because these conditions represented the most divergent infection phenotypes in our experimental system. These analyses identified broad response patterns associated with epidemiologically derived associations and infection outcomes: higher predicted compatibility pairs, which also showed higher bacterial burdens (L5 in THP-1m and *M. bovis* in BoMac), were associated with replication and cell-cycle signatures, whereas lower predicted compatibility pairs, which corresponded to lower-burden combinations, showed stronger endosomal, antigen-presentation and immune/containment-associated programmes. In addition, the differential exon-usage analysis in THP-1m highlighted strain-associated differences in pathways related to FcγR-mediated phagocytosis and actin/cytoskeleton remodelling, including cofilin-associated signals, whereas ferroptosis-related enrichment was observed in *M. bovis*-associated transcriptional signatures. We therefore interpret these pathways as candidate mechanisms associated with strain-dependent infection phenotypes, rather than as functionally validated drivers. Dedicated perturbation experiments would be required to determine whether modulation of these pathways directly alters intracellular burden, cell death or infection outcome. Within the macrophage model in which each strain achieved higher infection outcomes, we observed transcriptional patterns consistent with reduced activation of antimicrobial containment processes, including pathways linked to antigen presentation (MHC II), endosomal and vesicular organization and cellular compartmentalization. These processes have been implicated in immune recognition and intracellular control of *M. tuberculosis*, and their attenuation may facilitate immune evasion and persistence in macrophages [45, 46]. In parallel, conditions associated with higher infection phenotypes showed induction of genes linked to host cell proliferation and DNA replication, which may reflect a cellular state associated with higher intracellular burden. A comparable strategy has been described in other intracellular infections, including *Chlamydia trachomatis*, where manipulation of host proliferation contributes to persistence [47].

Conversely, strain–model combinations associated with less compatible epidemiological pairs, which showed lower infected-cell frequencies and lower intracellular burden, displayed a distinct transcriptional profile enriched for intracellular containment. Host cells upregulated compartmentalization pathways and genes involved in cellular defence and degradation of intracellular pathogens (for example *UBXN6*, *ABCD4*, *TFEB*; Table S11). The relevance of these mechanisms in *M. tuberculosis* infection is supported by studies showing that robust phagosome/endosome compartmentalization and immune activation can restrict bacterial growth and contribute to clearance [48]. In addition to these shared containment-associated signatures, we observed strain-specific behaviours. For example, *M. bovis* infection was associated with enrichment of ferroptosis-related pathways relative to L5. Ferroptosis, characterized by iron-dependent lipid peroxidation, has been reported as an important necrotic mechanism during *M. tuberculosis* infection *in vitro* and *in vivo* [49].

To prioritize candidate pathways that are induced upon infection but reduced in conditions associated with higher intracellular burden within each macrophage model, we selected genes that were upregulated in at least one infected condition relative to uninfected controls yet downregulated in the higher-burden strain relative to the alternative strain in that model. This approach highlighted genes and pathways linked to Golgi vesicle transport, peroxisomal function, NFκB and IL-17 signalling, ferroptosis, T cell activation and hypoxia responses. Their targeted reduction in conditions associated with higher intracellular infection phenotypes is consistent with a strategy in which more permissive infections attenuate immune and metabolic stress programmes [49, 50]. Such suppression could facilitate intracellular persistence by impairing vesicle trafficking, immune signalling and stress responses [51]. In more restrictive conditions, these pathways were not reduced, plausibly enabling more effective host protection. Within the proposed conceptual framework, this pattern is consistent with the idea that compatibility may reflect, in part, the degree to which a strain can attenuate host programmes that would otherwise contribute to containment. The different immune repertoire of BoMac [39, 40, 44] is likely to reduce the response of this cell line to infection, and might result in an underestimation of pathways that are common to highly compatible combinations in both cell types. We also observed differences in the temporal progression of host programmes between conditions associated with preferred vs unpreferred epidemiological matches. At early time points, conditions associated with higher intracellular burden showed enrichment of processes linked to cellular reorganization, metabolism and ribosome biogenesis, together with reduced apoptotic signalling and increased adhesion-associated pathways. At later stages, these conditions exhibited enrichment of pathways modulating lysosomal function and autophagosome formation, which may favour intracellular persistence [52] and is known to influence *M. tuberculosis* control [53]. In addition, enrichment of glycan degradation and polysaccharide metabolism suggests a shift toward resource acquisition strategies that are consistent with evidence that *M. tuberculosis* can access glucose *in vivo* and rely on glucose phosphorylation during chronic infection [54]. By contrast, conditions associated with lower infection outcomes showed earlier enrichment of oxygen-level modulation and oxidative stress responses, potentially consistent with antimycobacterial roles of peroxisomes in human macrophages [55], together with enhanced vesicle transport mechanisms in endosomal compartments that may support bacterial control [56].

Beyond gene-level changes, alternative splicing is an additional layer of host response that can shape infection phenotypes. Previous work has highlighted the importance of alternative splicing during *in vitro* infection with virulent and avirulent laboratory *M. tuberculosis* strains [57]. However, systematic comparisons of splicing across *B. taurus* and *H. sapiens* are complicated by differences in reference genome and annotation quality [58]. For this reason, DEU analyses were restricted to human annotations. In THP-1m infections, we observed hundreds of genes displaying strain-associated isoform usage patterns. While functional impacts remain to be established, several cases were notable. *IL-16* exhibited distinct isoform usage between strains, with multiple exon-usage events linked to conditions associated with lower infection outcomes. Because *IL-16* functions as a chemoattractant and modulator of T cell activation [59], isoform differences could plausibly influence immune signalling and downstream inflammatory responses.

We also detected DEU within pathways linked to Fc gamma R-mediated phagocytosis, together with gene-level differences that may further clarify how strains elicit divergent macrophage programmes. Among these, *cofilin-2* was upregulated in both human and bovine macrophage models under conditions associated with higher infection outcomes and has been implicated in regulating phagocytosis and phagosome-lysosome fusion in bone-marrow-derived macrophages [43, 60]. This is consistent with its established role in actin cytoskeleton remodelling, a process that directly influences macrophage engulfment and intracellular trafficking [61]. Because cofilin regulates actin polymerization dynamics, invasive pathogens can exploit this axis [43, 62], and disruptions in actin polymerization can impair phagosome-lysosome fusion in other intracellular infections [60]. In contrast, conditions associated with lower infection outcomes showed increased *SPHK* expression, a factor implicated in intracellular signalling and phagosome maturation that can promote phagosome-lysosome fusion and bacterial clearance [42]. *SPHK*-1 can restrict *Mycobacterium smegmatis* in macrophages [63] and has been shown to control pathogenic *M. tuberculosis* in a murine model [64]. Together, these observations are consistent with the hypothesis that actin and phagosome maturation pathways are differentially engaged across strain–model combinations, although direct perturbation of these pathways will be required to establish their functional contribution to infection outcome.

Genomic differences between human- and animal-associated MTBC ecotypes may contribute to host association and differential infection outcomes. One notable difference is RD5, which has been repeatedly lost in animal-associated lineages and includes PPE genes together with the *plcABC* region; in contrast, *plcD* is retained outside RD5 [65, 66]. Consistent with this, we observed substantial lineage-level variation in *plc* transcription across infections. Moreover, *plc* transcription varied with macrophage context across infections, consistent with the idea that an intracellular environment can shape plc transcriptional output. Negligible *plcC* expression in L6 at 1 dpi may relate to the non-synonymous mutation in *plcC* (ENA accession id: ERR2704688). Prior work has shown that *plc* genes can inhibit *PTGER2* and *PTGER4* [25], receptors that participate in immune responses and inflammation through the eicosanoid pathway [67]. In our data, higher *PTGER* levels were observed in conditions associated with lower infection outcomes, which may reflect a more robust defensive response. In addition, opposite association patterns between *plcC* and *PTGER* expression across THP-1m and BoMac highlight how macrophage context can influence the interaction between virulence-linked bacterial pathways and host receptors. U73122/D609 treatment reduced intracellular c.f.u. and LDH release in THP-1m infections with both L5 and *M. bovis*, whereas no significant effect was detected in BoMac infections with either strain. Thus, inhibitor sensitivity was associated with macrophage context rather than with bacterial strain identity or *plcC* transcript status. Because *M. bovis* just expresses *plcD*, the inhibitor effect observed in *M. bovis*-infected THP-1m can be attributed to the presence of *plcD* or the presence of only one *plc* gene. Conversely, the lack of inhibitor effect in BoMac despite detectable L5 *plcC* expression indicates that *plcC* transcript abundance alone does not predict inhibitor sensitivity across macrophage models.

We analysed a restricted set of MTBC strains (human-associated L5 and L6, plus animal-associated chimpanzee bacillus and *M. bovis*), which does not capture the full diversity of MTBC. Substantial variation is expected among strains and lineages [68, 69], and expanding the number of strains per lineage will be important to generalize lineage-level trends. Moreover, THP-1m and BoMac are immortalized *in vitro* models that can differ from primary macrophages in signalling and functional characteristics [70], which limit direct extrapolation to *in vivo* host specificity. Stevens and colleagues emphasize that macrophage origin can strongly shape inflammatory outputs during mycobacterial infection even when bacterial growth control is comparable [71]. Studies using primary macrophages address how cell-line findings relate to primary cell systems [34, 35]. Despite the limitation of using cell lines, our results are consistent with the work reported by Queval *et al*. [35], who used primary cells. They reported that *M. tuberculosis* and *M. bovis* can exhibit comparable replication patterns in bovine macrophages during early infection, whereas differences are more evident in human macrophages. Similarly, in our cell-line models, *M. bovis* and L5 showed broadly comparable normalized c.f.u. expansion over the 72 h infection window, and apoptosis/necrosis readouts were not markedly different between these two strains within either macrophage model. This point is consistent with prior work by Steven *et al*. [71] showing that macrophages of different origin can mount distinct inflammatory responses to mycobacterial infection even when bacterial growth control is comparable [71]. Accordingly, we present this model as an experimentally tractable framework to explore host specificity. By comparing the relative effects of different bacterial strains in two macrophage models, we show that host–pathogen ‘compatibility’ can be treated as a quantitative parameter linking infection phenotypes to host transcriptional programmes. This framework provides a basis for deciphering host-specific infection signatures and motivates future work to test how these differences translate into *in vivo* host range and evolutionary adaptation.

## Supplementary material

10.1099/mgen.0.001826 Fig. S1.

10.1099/mgen.0.001826 Fig. S2.

10.1099/mgen.0.001826 Fig. S3.

10.1099/mgen.0.001826 Fig. S4.

10.1099/mgen.0.001826 Fig. S5.

10.1099/mgen.0.001826 Table S1.

10.1099/mgen.0.001826 Table S2.

10.1099/mgen.0.001826 Table S3.

10.1099/mgen.0.001826 Table S4.

10.1099/mgen.0.001826 Table S5.

10.1099/mgen.0.001826 Table S6.

10.1099/mgen.0.001826 Table S7.

10.1099/mgen.0.001826 Table S8.

10.1099/mgen.0.001826 Table S9.

10.1099/mgen.0.001826 Table S10.

10.1099/mgen.0.001826 Table S11.

10.1099/mgen.0.001826 Table S12.

10.1099/mgen.0.001826 Table S13.

10.1099/mgen.0.001826 Table S14.

10.1099/mgen.0.001826 Table S15.

10.1099/mgen.0.001826 Table S16.

10.1099/mgen.0.001826 Table S17.

10.1099/mgen.0.001826 Table S18.

## Abbreviations

BCG: Bacillus Calmette–Guérin
DEG: differentially expressed gene
DEOG: differentially expressed one-to-one orthologue
DEU: differential exon usage
dpi: day post-infection
ECM: extracellular matrix
FCS: Flow Cytometry Standard
GO: Gene Ontology
GOEA: Gene Ontology enrichment analysis
hpi: hour post-infection
IMDM: Iscove’s Modified Dulbecco’s Medium
IQR: interquartile range
KEA: KEGG pathway enrichment analysis
KEGG: Kyoto Encyclopedia of Genes and Genomes
LDH: lactate dehydrogenase
MAPK: mitogen-activated protein kinase
MTBC: *Mycobacterium tuberculosis* complex
OADC: oleic acid–albumin–dextrose–catalase
PMA: phorbol 12-myristate 13-acetate
PPE: Pro–Pro–Glu
PTGER: prostaglandin E receptor
RPMI: Roswell Park Memorial Institute
