# Supplementary material for: Host–strain compatibility shapes host transcriptional responses in macrophage in vitro infection models of Mycobacterium tuberculosis
Source: Microb Genom. 2026 Sep 23;12(9):001826. doi: 10.1099/mgen.0.001826 (PMC13600463; doi:10.1099/mgen.0.001826)
Supplement: Supplementary file 1 [file mgen-12-01826-s001.pdf]

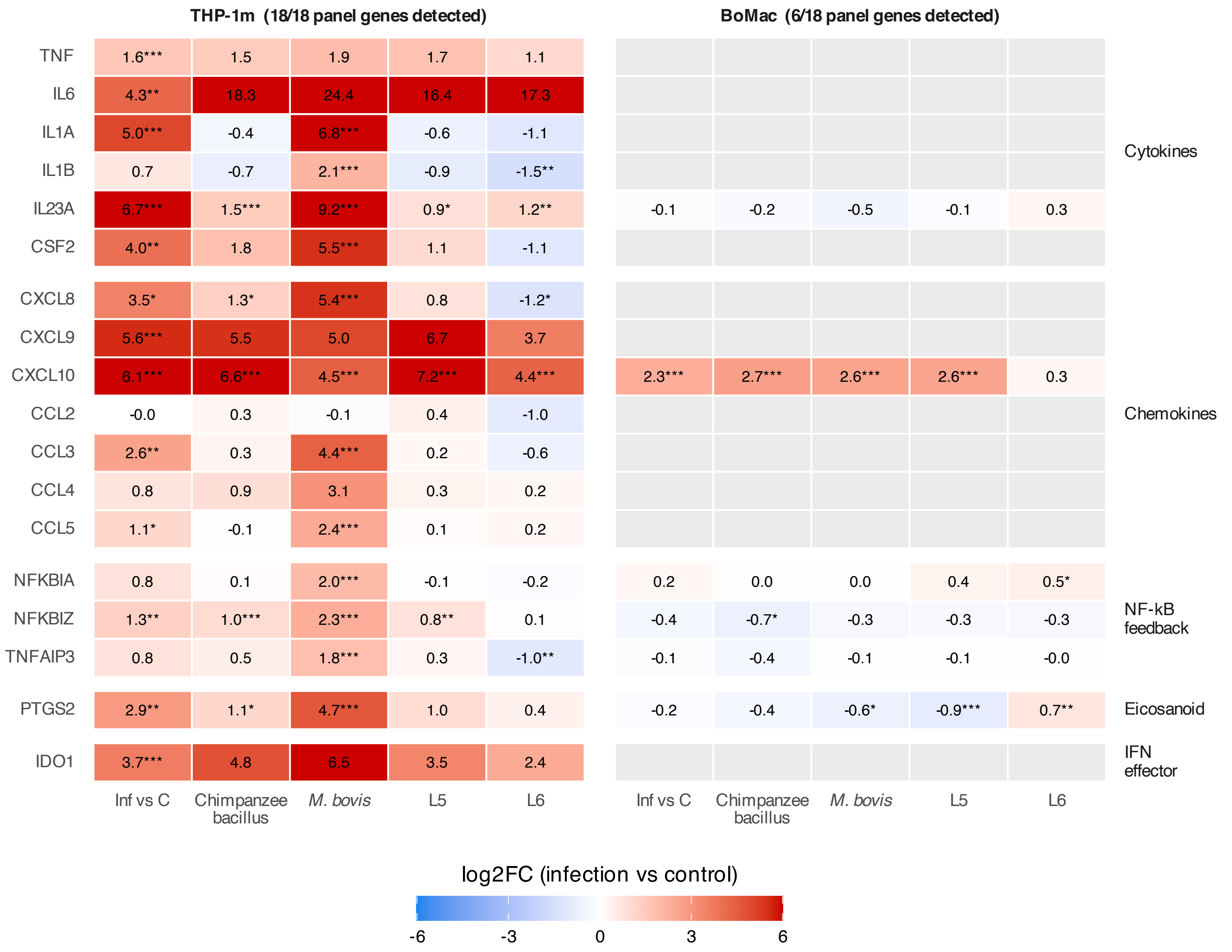

log2FoldChange (DESeq2, both dpi pooled). Stars: * padj<0.05 ** <0.01 *** <0.001. Grey = below detection (not tested).
