# Supplementary material for: Host–strain compatibility shapes host transcriptional responses in macrophage in vitro infection models of Mycobacterium tuberculosis
Source: Microb Genom. 2026 Sep 23;12(9):001826. doi: 10.1099/mgen.0.001826 (PMC13600463; doi:10.1099/mgen.0.001826)
Supplement: Supplementary file 2 [file mgen-12-01826-s002.pdf]

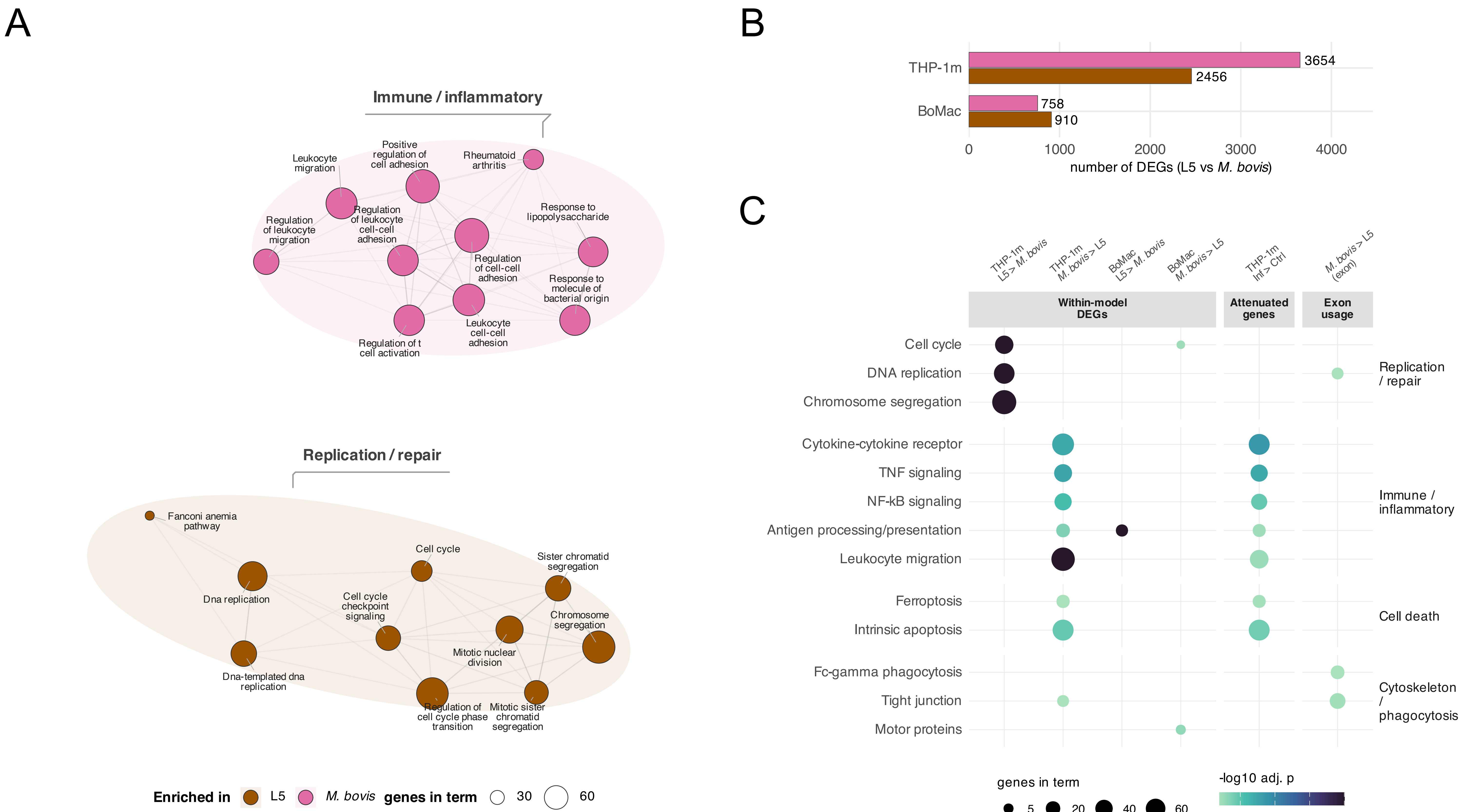
A
Immune / inflammatory
Leukocyte migration
Positive regulation of cell adhesion
Rheumatoid arthritis
Regulation of leukocyte migration
Regulation of leukocyte cell-cell adhesion
Response to lipopolysaccharide
Regulation of cell-cell adhesion
Response to molecule of bacterial origin
Leukocyte cell-cell adhesion
Regulation of t cell activation
Replication / repair
Fanconi anemia pathway
Cell cycle
Sister chromatid segregation
Dna replication
Cell cycle checkpoint signaling
Chromosome segregation
Mitotic nuclear division
Dna-templated dna replication
Regulation of cell cycle phase transition
Mitotic sister chromatid segregation
Enriched in
L5
M. bovis
genes in term
30
60
B
THP-1m
BoMac
3654
2456
758
910
0
1000
2000
3000
4000
number of DEGs (L5 vs M. bovis)
C
THP-1m L5 > M. bovis
THP-1m M. bovis > L5
BoMac L5 > M. bovis
BoMac M. bovis > L5
THP-1m Inf > Ctrl
M. bovis > L5 (exon)
Within-model DEGs
Attenuated genes
Exon usage
Cell cycle
DNA replication
Chromosome segregation
Replication / repair
Cytokine-cytokine receptor
TNF signaling
NF-kB signaling
Antigen processing/presentation
Leukocyte migration
Immune / inflammatory
Ferroptosis
Intrinsic apoptosis
Cell death
Fc-gamma phagocytosis
Tight junction
Motor proteins
Cytoskeleton / phagocytosis
genes in term
5
20
40
60
-log10 adj. p
5
10
15
>=20
