# Supplementary material for: Host–strain compatibility shapes host transcriptional responses in macrophage in vitro infection models of Mycobacterium tuberculosis
Source: Microb Genom. 2026 Sep 23;12(9):001826. doi: 10.1099/mgen.0.001826 (PMC13600463; doi:10.1099/mgen.0.001826)
Supplement: Supplementary file 3 [file mgen-12-01826-s003.pdf]

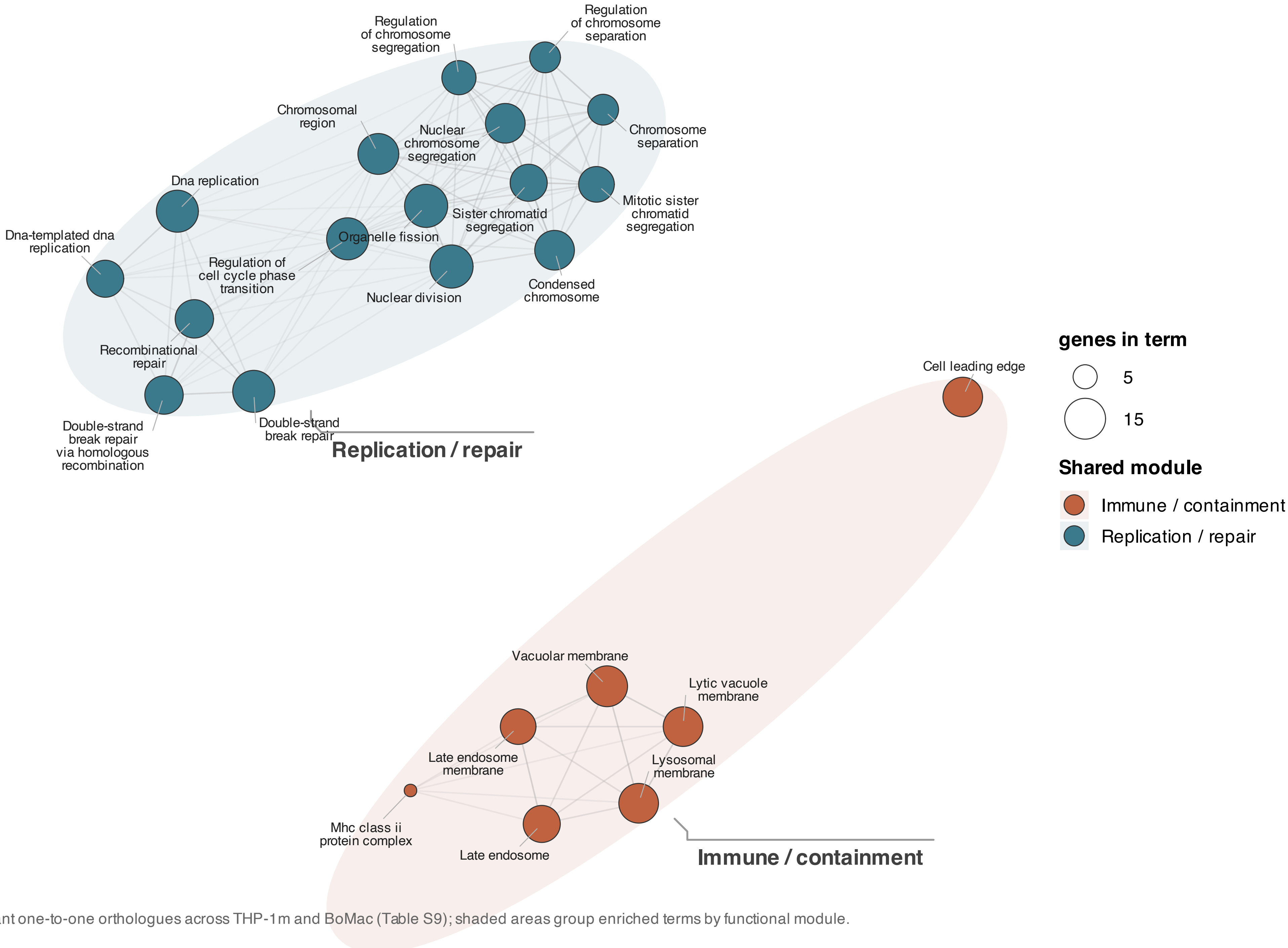

Concordant one-to-one orthologues across THP-1m and BoMac (Table S9); shaded areas group enriched terms by functional module.
