# Supplementary material for: Host–strain compatibility shapes host transcriptional responses in macrophage in vitro infection models of Mycobacterium tuberculosis
Source: Microb Genom. 2026 Sep 23;12(9):001826. doi: 10.1099/mgen.0.001826 (PMC13600463; doi:10.1099/mgen.0.001826)
Supplement: Supplementary file 4 [file mgen-12-01826-s004.pdf]

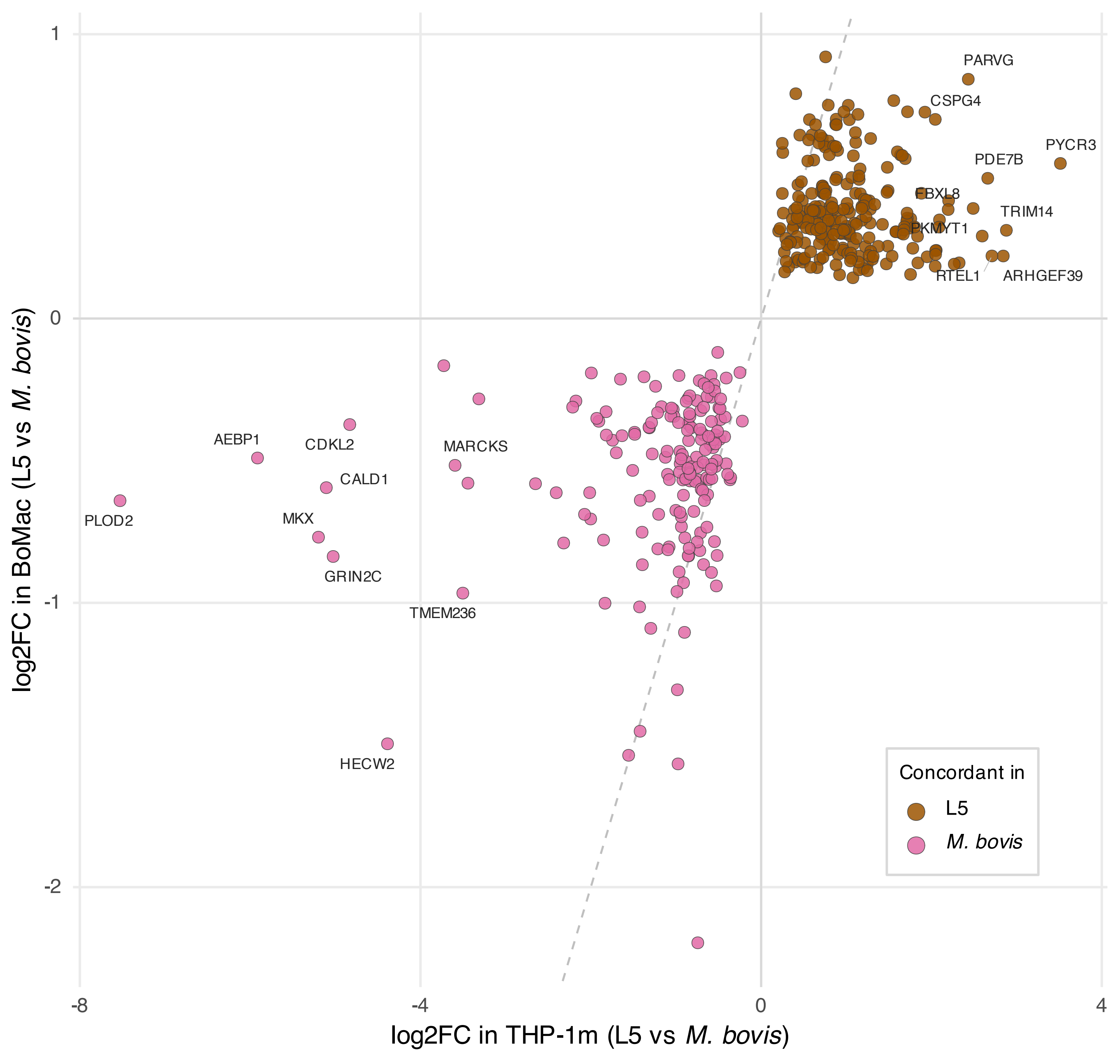

1
0
-1
-2
-8
-4
0
4
log2FC in BoMac (L5 vs *M. bovis*)
log2FC in THP-1m (L5 vs *M. bovis*)
PARVG
CSPG4
PYCR3
PDE7B
FBXL8
TRIM14
PKMYT1
RTEL1
ARHGEF39
AEBP1
CDKL2
MARCKS
CALD1
PLOD2
MKX
GRIN2C
TMEM236
HECW2
Concordant in
L5
*M. bovis*
